# Prognostic inflammatory–immune score-based risk stratification optimizes adjuvant therapy for non-gastric gastrointestinal stromal tumors: a multicenter study

**DOI:** 10.3389/fimmu.2026.1846854

**Published:** 2026-06-02

**Authors:** Zhiming Cai, Zhengnan Xu, Huimei Lin, Huibin Liu, Zihan Lin, Jinhu Chen, Shichai Hong, Weibin Song, Xinyu Chen, Yanchang Xu, Zhenrong Yang, Yongjian Zhou

**Affiliations:** 1 Department of Gastrointestinal Surgery, The First Affiliated Hospital, Fujian Medical University, Fuzhou, China; 2 Department of Gastrointestinal Surgery, National Regional Medical Center, Binhai Campus of the First Affiliated Hospital, Fujian Medical University, Fuzhou, China; 3 Department of Gastrointestinal Surgery, The First Hospital of Putian City, Putian, China; 4 Department of Anorectal Surgery, The Second Affiliated Hospital of Xiamen Medical College, Xiamen, China; 5 Department of Gastrointestinal Surgery, The Affiliated Hospital of Putian University, Putian, China; 6 Department of Gastric Surgery, Fujian Medical University Union Hospital, Fuzhou, China

**Keywords:** adjuvant therapy, gastrointestinal stromal tumor, inflammatory-immune score, machine learning, nomogram, non-gastric

## Abstract

**Background:**

Conventional modified National Institutes of Health (mNIH) and Armed Forces Institute of Pathology (AFIP) systems exhibit “anatomical bias” due to their development from predominantly gastric cohorts, resulting in suboptimal prognostic performance in non-gastric gastrointestinal stromal tumors (GISTs). This study aimed to develop an integrated model to refine risk stratification and guide adjuvant imatinib duration in non-gastric GIST.

**Methods:**

A multicenter retrospective cohort of 471 patients with non-gastric GIST was analyzed. Hematological variables were screened using the Boruta algorithm to construct a Prognostic Inflammatory–Immune Score (PIIS), which was integrated with clinicopathological factors to develop a nomogram. Feature contributions were interpreted using Shapley Additive Explanations (SHAP). Risk re-stratification was conducted to evaluate benefits of different adjuvant imatinib durations.

**Results:**

The PIIS comprised the platelet-to-albumin ratio (PAR), platelet-to-lymphocyte ratio (PLR), derived neutrophil-to-lymphocyte ratio (dNLR), and lactate dehydrogenase-to-albumin ratio (LAR). Multivariable analysis identified sex, tumor size, mitotic index, Ki-67 index, and PIIS as independent predictors of recurrence-free survival (RFS). The integrated model demonstrated superior discrimination, with C-indices of 0.839 in the training cohort and 0.795 in the external validation cohort, outperforming mNIH and AFIP. The model revealed substantial heterogeneity within conventional risk categories and stratified mNIH high-risk and AFIP intermediate- and high-risk patients into low-, medium-, and high-risk groups. In model-defined medium- and high-risk subgroups, adjuvant therapy beyond 3 years was associated with improved RFS, whereas no clear additional benefit was observed in the low-risk subgroup.

**Conclusions:**

This multicenter-validated nomogram may improve recurrence risk prediction in non-gastric GIST and may provide a useful reference for risk-adapted adjuvant therapy planning.

## 1 Introduction

Gastrointestinal stromal tumors (GISTs) are the most common mesenchymal neoplasms of the gastrointestinal tract and exhibit marked biological heterogeneity (1, 2). Although approximately 60% of GISTs arise in the stomach, non-gastric GISTs differ substantially from gastric GISTs in terms of clinicopathological characteristics and prognosis (3). Accumulating evidence indicates that, compared with primary gastric GISTs, non-gastric tumors generally display more aggressive biological behavior, a higher risk of postoperative recurrence, and poorer overall outcomes (4–6). Several risk stratification systems have been developed to estimate postoperative recurrence risk and guide adjuvant treatment decisions in GIST, including the modified National Institutes of Health (mNIH) criteria and the Armed Forces Institute of Pathology (AFIP) classification (7, 8), among which the mNIH system is the most widely applied in clinical practice. However, the mNIH criteria was primarily derived from cohorts dominated by gastric GISTs, and its applicability to non-gastric GIST remains challenging. Its prognostic accuracy in low-risk patients has been questioned (9), while the intermediate-risk category is rarely represented in non-gastric GIST, resulting in a marked gap in risk stratification and limiting the discriminatory capacity of the system (10, 11). Consequently, the identification of novel biomarkers and risk assessment tools capable of more accurately predicting prognosis in non-gastric GIST is of considerable clinical importance.

Accumulating evidence indicates that tumor progression is determined not only by intrinsic tumor characteristics but also by the host immune and inflammatory status within the tumor microenvironment (12–14). In this context, several peripheral blood–based inflammatory and immune indices, such as the derived neutrophil-to-lymphocyte ratio, platelet-to-lymphocyte ratio, and lactate dehydrogenase levels, have been proposed as surrogate markers of tumor-associated immune–inflammatory responses and have demonstrated prognostic value across a wide range of malignancies (15–18). Nevertheless, most existing studies have focused on single inflammatory markers, and few have systematically evaluated their prognostic relevance specifically in non-gastric GIST, a distinct high-risk subgroup (19). Single biomarkers are susceptible to physiological fluctuations and comorbid conditions and therefore may fail to comprehensively capture the complexity of the host immune–inflammatory milieu. Meanwhile, advances in artificial intelligence and biostatistics have enabled machine learning algorithms to integrate multidimensional heterogeneous data, thereby improving model robustness and interpretability (20, 21).

In this study, we leveraged a multicenter retrospective cohort from five hospitals in China and applied a rigorous machine learning strategy to identify and construct a novel Prognostic Inflammatory–Immune Score (PIIS). By further integrating key clinicopathological variables, we developed and validated an individualized nomogram for predicting postoperative recurrence risk in patients with non-gastric GIST. Importantly, we also evaluated the clinical utility of this model in guiding prolonged adjuvant imatinib therapy among high-risk patients, with the aim of providing evidence to support precision-driven clinical decision-making in non-gastric GIST.

## 2 Materials and methods

### 2.1 Patients

**Figure 1:**
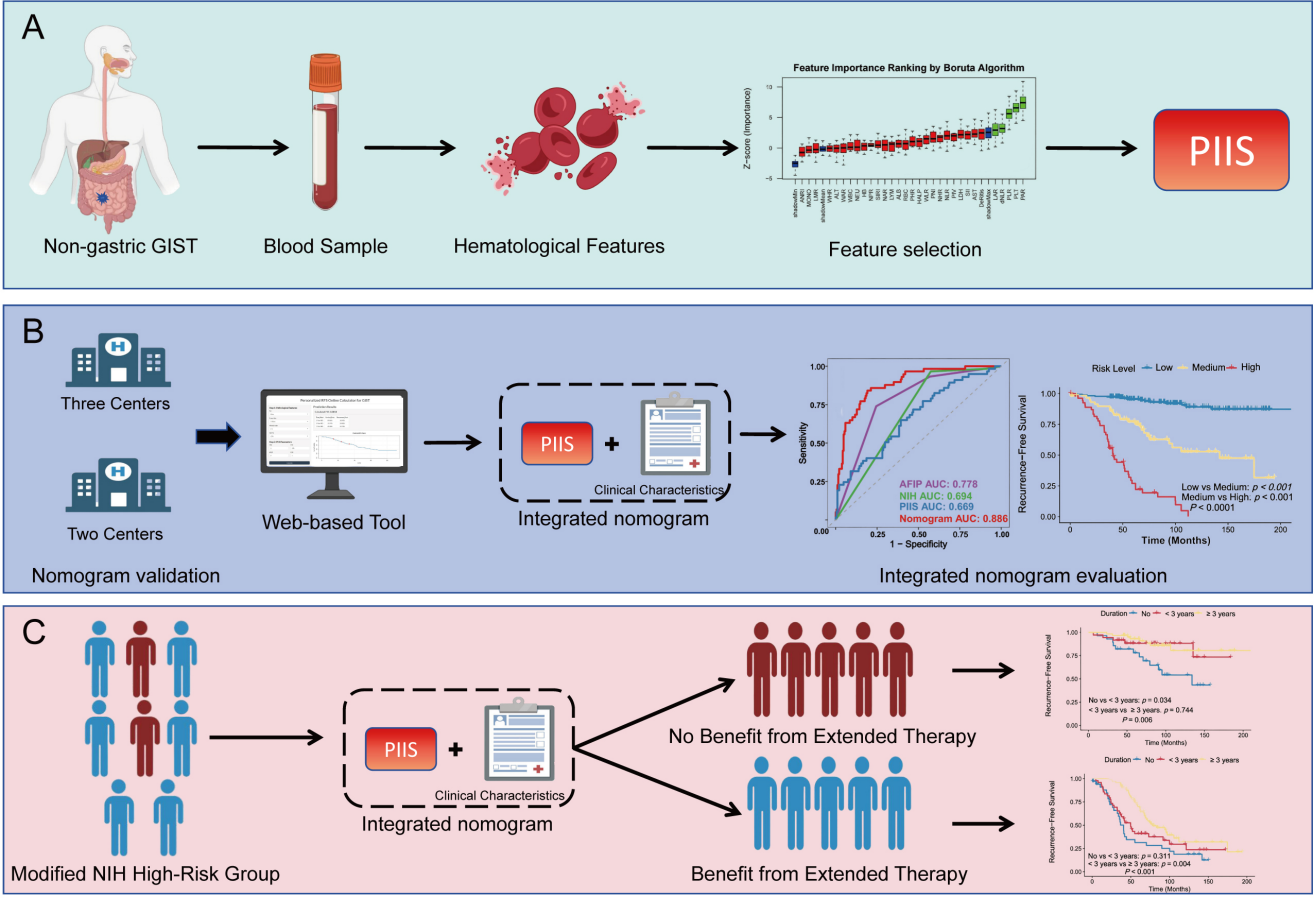
Study workflow. **(A)** Feature selection using the Boruta algorithm to construct the Prognostic Inflammatory-Immune Score (PIIS). **(B)** Nomogram development and multicenter validation. **(C)** Assessment of clinical utility in the modified NIH high-risk group.

The study design is shown in **Figure 1**. This retrospective analysis consecutively enrolled 471 patients with primary non-gastric GISTs who underwent curative resection between March 2007 and December 2022 at five hospitals in China. The development (n=297) and external validation (n=174) cohorts were derived from three and two independent centers, respectively. The inclusion criteria were as follows: (1) histopathologically confirmed primary non-gastric GIST; (2) no history of other malignancies; and (3) achievement of R0 resection. The exclusion criteria were: (1) recurrent or metastatic disease; (2) receipt of neoadjuvant therapy; (3) perioperative mortality; and (4) incomplete clinical or follow-up data. The patient enrollment process is detailed in **Supplementary Figure 1**.

### 2.2 Data collection and definitions

Demographic and clinicopathological data were extracted from the electronic medical record system, including age, sex, tumor site, tumor size, mitotic index, Ki-67 index, tumor rupture, tumor necrosis, duration of postoperative adjuvant therapy, the 2008 mNIH and AFIP classification. Tumor site was categorized as non-gastric gastrointestinal or extragastrointestinal. Non-gastric gastrointestinal GISTs originated from the gastrointestinal tract excluding the stomach, while extragastrointestinal GISTs arose outside the tubular gastrointestinal tract, including the mesentery, omentum, retroperitoneum and pelvis. Peripheral blood samples were obtained within 2 weeks before surgery. Laboratory variables included white blood cells (WBC), neutrophils (NEU), platelets (PLT), red blood cells (RBC), monocytes (MONO), lymphocytes (LYM), hemoglobin (HB), albumin (ALB), lactate dehydrogenase (LDH), aspartate aminotransferase (AST), and alanine aminotransferase (ALT). Based on combinations of these parameters, 20 composite inflammatory indices were calculated: PLT-to-ALB ratio (PAR), PLT-to-HB ratio (PHR), PLT-to-LYM ratio (PLR), WBC-to-ALB ratio (WAR), WBC-to-HB ratio (WHR), WBC-to-LYM ratio (WLR), NEU-to-ALB ratio (NAR), NEU-to-HB ratio (NHR), NEU-to-LYM ratio (NLR), derived NEU-to-LYM ratio (dNLR), systemic immune–inflammation index (SII; PLT × NEU/LYM), systemic inflammation response index (SIRI; NEU × MONO/LYM), prognostic nutritional index (PNI; ALB + 5 × LYM), LYM-to-MONO ratio (LMR), NEU-to-PLT ratio (NPR), prognostic inflammatory value (PIV; PLT × NEU × MONO/LYM), HB–ALB–LYM–PLT index (HALP; HB × ALB × LYM/PLT), LDH-to-ALB ratio (LAR), De Ritis ratio (AST/ALT), and AST-to-NEU ratio (ANRI).

### 2.3 Data preprocessing

Missing hematological data were imputed using the predictive mean matching (PMM) algorithm within the multiple imputation framework via the mice package. To integrate survival information, the imputation model incorporated the Nelson–Aalen cumulative hazard estimator and recurrence status. The imputation model was executed for 50 iterations, generating 5 imputed datasets. To reduce inter-center batch effects related to heterogeneous detection platforms and laboratory equipment, the ComBat algorithm from the sva package was applied to log-transformed hematological variables (**Supplementary Figure 2**). All continuous variables were subsequently standardized using Z-score normalization for downstream analyses.

### 2.4 Construction of the PIIS

The PIIS was developed using a rigorous three-step feature selection strategy to identify robust and independent inflammatory–immune prognostic predictors from 31 candidate hematological variables. First, the random forest–based Boruta algorithm using the Boruta package was applied to perform initial screening, setting a maximum of 100 iterations (maxRuns=100) and applying the TentativeRoughFix function to classify tentative variables. This algorithm compares the importance of original variables with randomly permuted shadow features, retaining those with consistently higher importance than the shadow variables, thereby preliminarily locking in a key candidate set. Second, Spearman correlation analysis was conducted to visualize inter-variable associations and identify highly correlated feature clusters. Third, to eliminate the impact of multicollinearity on model stability, the car package was utilized to calculate the variance inflation factors (VIFs) of the remaining candidate variables, and variables with VIF > 5 were iteratively excluded to ensure sufficient independence of each predictor incorporated into the final model. The remaining independent variables were subsequently entered into a multivariable Cox proportional hazards model, and the resulting regression coefficients were used to construct the linear PIIS.

### 2.5 Interpretability analysis

To improve interpretability of the multivariable prognostic model, SHapley Additive exPlanations (SHAP) were applied to attribute model predictions (22). Specifically, an XGBoost algorithm was utilized to fit the Cox survival objective function as a surrogate model, and the shapviz package was employed to calculate and visualize the contribution (Shapley value) of each feature to the final risk score for individual patients. This approach enables evaluation of the relative importance of clinicopathological variables and the PIIS at the global level, while intuitively illustrating how each feature positively or negatively influences recurrence risk at the individual level.

### 2.6 Nomogram construction and validation

Independent prognostic factors for recurrence-free survival (RFS) identified by univariable and multivariable Cox proportional hazards analyses in the training cohort were integrated to construct a nomogram using the rms package. Its performance was compared with that of the PIIS, mNIH, and AFIP systems in both the training and validation cohorts across multiple dimensions. Discrimination was assessed using the concordance index (C-index) via the survival package, time-dependent area under the curve (AUC) via the timeROC package, and integrated AUC (iAUC) based on Bootstrap resampling. Calibration was evaluated via calibration curves (comparing predicted probabilities from survival package-fitted Cox models with actual Kaplan-Meier estimates using quartile grouping at 3-, 5-, and 7-year intervals) and the Brier score calculated via the pec package. Goodness-of-fit was determined by the Akaike and Bayesian information criteria (AIC and BIC) using the coxphf package based on Firth’s penalized Cox regression. Clinical net benefit was assessed using decision curve analysis (DCA). For each model, time-dependent recurrence risk estimates were derived from Cox models fitted using the survival package and were used to calculate net benefit across a range of threshold probabilities, with treat-all and treat-none strategies as references. DCA curves were generated using custom R code and visualized with the ggplot2 package. The proportional hazards assumption was evaluated using the cox.zph function based on Schoenfeld residuals. Optimal risk stratification cutoffs for the total nomogram scores were identified using X-tile software (v3.6.1), and within conventionally defined high-risk patients, the differential benefit of prolonged adjuvant therapy across risk strata was explored.

### 2.7 Statistical analysis

All statistical analyses were conducted using R software (version 4.2.2). Normally distributed continuous variables are presented as mean ± standard deviation and were compared using independent-samples t tests, whereas non-normally distributed variables are reported as median [interquartile range (IQR)] and compared using the Mann–Whitney U test. Categorical variables are expressed as frequencies (percentages) and compared using the chi-square or Fisher’s exact test, as appropriate. Survival outcomes were analyzed using the Kaplan–Meier method, with between-group differences assessed by the log-rank test. All tests were two-sided, and P < 0.05 was considered statistically significant.

## 3 Results

### 3.1 Baseline characteristics

A total of 471 patients with primary non-gastric GIST were enrolled, including 297 in the training cohort and 174 in the external validation cohort. The cohorts were well balanced across most characteristics, including age, sex, tumor site and size, tumor necrosis, Ki-67 index, tumor rupture, gene mutation status, and modified NIH and AFIP classifications (all P > 0.05). A significant difference was observed only in the mitotic index (P < 0.001; **Table 1**). Survival outcomes were comparable between cohorts (**Supplementary Figure 3**), and the median follow-up for the entire population was 85 (IQR, 58–120) months.

**Table 1:** Baseline demographic and clinical characteristics of patients.

Characteristic	Total	Training	Validation	*P*-value
	n=471	n=297	n=174	
Age (years)				0.902
<60	267 (56.7)	169 (56.9)	98 (56.3)	
≥60	204 (43.3)	128 (43.1)	76 (43.7)	
Sex				0.079
Male	235 (49.9)	139 (46.8)	96 (55.2)	
Female	236 (50.1)	158 (53.2)	78 (44.8)	
Tumor site				0.452
Non-gastric gastrointestinal	404 (85.8)	252 (84.8)	152 (87.4)	
Extragastrointestinal	67 (14.2)	45 (15.2)	22 (12.6)	
Tumor size (cm)				0.611
≤5	191 (40.6)	122 (41.1)	69 (39.7)	
>5 and ≤10	166 (35.2)	100 (33.7)	66 (37.9)	
>10	114 (24.2)	75 (25.2)	39 (22.4)	
Tumor necrosis				0.556
No	317 (67.3)	197 (66.3)	120 (69.0)	
Yes	154 (32.7)	100 (33.7)	54 (31.0)	
Mitotic index (/50HPF)				<0.001
≤2	227 (48.2)	126 (42.4)	101 (58.1)	
>2 and ≤5	140 (29.7)	106 (35.7)	34 (19.5)	
>5 and ≤10	56 (11.9)	39 (13.1)	17 (9.8)	
>10	48 (10.2)	26 (8.8)	22 (12.6)	
Ki67 (%)				0.226
≤5	343 (72.8)	215 (72.4)	128 (73.6)	
>5 and ≤10	54 (11.5)	30 (10.1)	24 (13.8)	
>10	74 (15.7)	52 (17.5)	22 (12.6)	
Tumor rupture				0.138
No	436 (92.6)	279 (93.9)	157 (90.2)	
Yes	35 (7.4)	18 (6.1)	17 (9.8)	
Gene Mutation				0.097
KIT exon 11	136 (28.9)	81 (27.3)	55 (31.6)	
Non-KIT exon 11	53 (11.2)	28 (9.4)	25 (14.4)	
Not tested	282 (59.9)	188 (63.3)	94 (54.0)	
Modified NIH criteria				0.718
Very low	39 (8.3)	23 (7.7)	16 (9.2)	
Low	125 (26.5)	82 (27.6)	43 (24.7)	
Intermediate	0 (0.0)	0 (0.0)	0 (0.0)	
High	307 (65.2)	192 (64.7)	115 (66.1)	
AFIP criteria				0.739
Very low	43 (9.1)	26 (8.8)	17 (9.8)	
Low	125 (26.5)	82 (27.6)	43 (24.7)	
Intermediate	142 (30.2)	85 (28.6)	57 (32.8)	
High	161 (34.2)	104 (35.0)	57 (32.7)	

The bold values indicate statistically significant P-values (P<0.05).

### 3.2 Construction and validation of the PIIS

Dimensionality reduction using the Boruta algorithm identified five features associated with RFS: PAR, PLR, dNLR, LAR, and PLT (**Supplementary Figure 4**). Subsequent collinearity diagnostics revealed generally weak correlations among most variables; however, PAR showed a strong correlation with PLT (r = 0.87), indicating substantial redundancy (**Supplementary Figure 5**). Consistently, VIF analysis demonstrated significant multicollinearity when all five variables were included, with several VIF values exceeding 5. After iterative removal of PLT, four independent predictors—PAR, PLR, dNLR, and LAR—were retained, all with VIF values below 5, ensuring model stability (**Supplementary Figure 6**).

Restricted cubic spline analyses were performed to characterize dose–response relationships between the four indicators and recurrence risk. In the training cohort, all indicators showed significant overall associations with RFS (P overall < 0.05). PLR and dNLR exhibited linear positive associations, whereas PAR and LAR showed significant nonlinear, monotonic increases in risk (P nonlinear < 0.05). Although the association between dNLR and RFS did not reach significance in the validation cohort, likely due to limited sample size, the direction of effect was consistent with that in the training cohort (**Supplementary Figure 7**).

Based on these four predictors, the PIIS was constructed using coefficients derived from a multivariable Cox model: PIIS = 0.0989 × PAR + 0.3053 × PLR + 0.0400 × dNLR + 0.2217 × LAR + 0.1435. The PIIS showed comparable distributions in the training and validation cohorts, indicating good transferability (**Supplementary Figure 8**).

Using an optimal cutoff, patients were stratified into high- and low-risk groups. Kaplan–Meier analyses demonstrated significantly different RFS between groups in both cohorts (**Supplementary Figure 9**). Subgroup analyses confirmed consistent risk discrimination across most clinicopathological strata, including age, sex, tumor size, and mitotic index. Although significance was not reached in the tumor rupture and non–KIT exon 11 mutation subgroups due to limited sample sizes, risk trends remained consistent (**Supplementary Figure 10**). Notably, low-risk patients defined by PIIS consistently achieved substantially higher 5-year RFS rates than those of high-risk patients across all subgroups, supporting the robustness and independent prognostic value of PIIS (**Supplementary Figure 11**).

### 3.3 Feature importance interpretation

**Figure 2:**
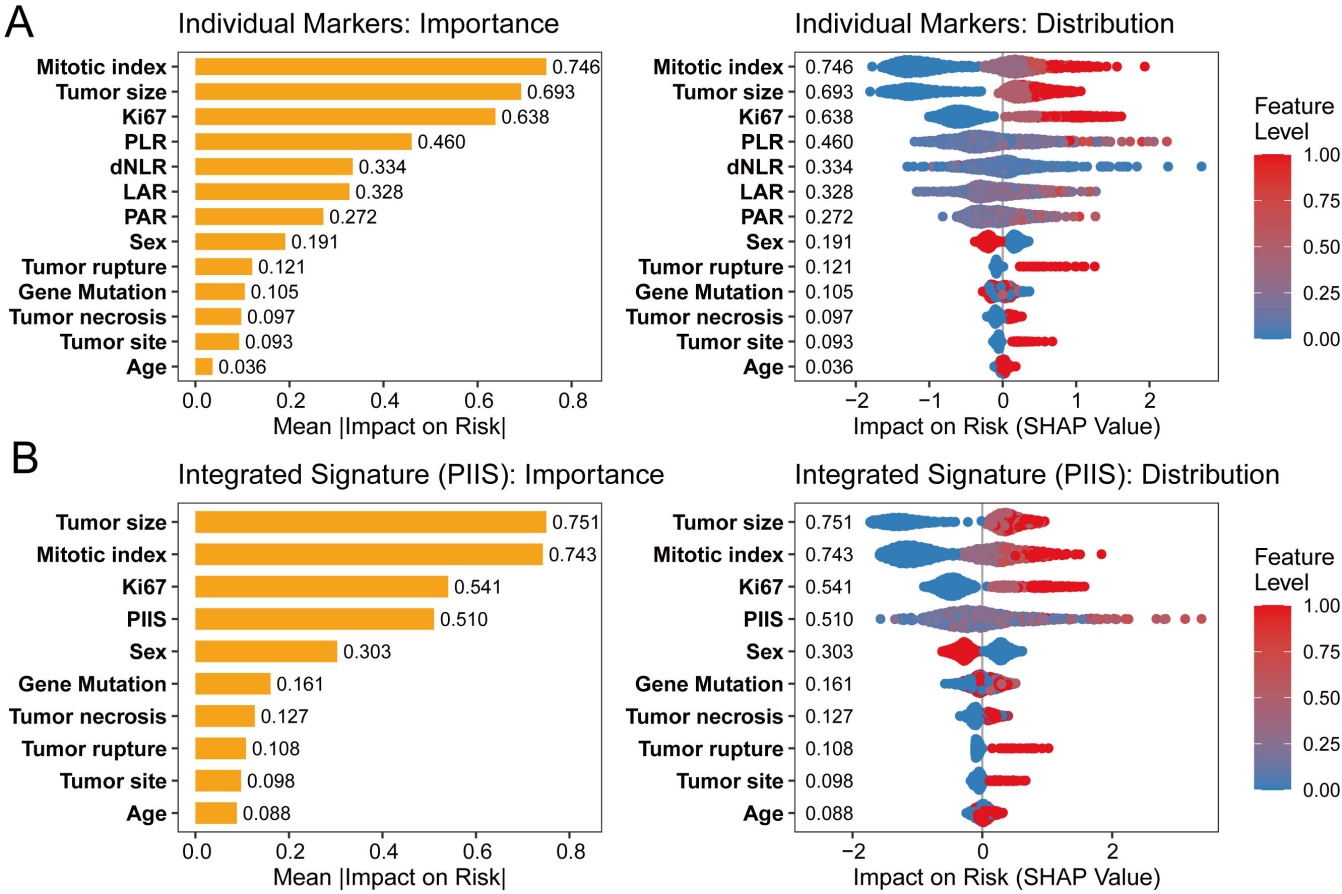
SHAP analysis showing the contributions of features to recurrence risk. **(A)** SHAP values for individual clinicopathological features and hematological markers (PLR, dNLR, PAR, and LAR). **(B)** SHAP values for the integrated Prognostic Inflammatory-Immune Score (PIIS) and clinicopathological characteristics.

To clarify the relative prognostic contributions of different feature categories, SHAP was used to compare the importance of individual hematological inflammatory markers, the integrated PIIS, and conventional clinicopathological variables. Global SHAP analysis showed that tumor size and mitotic index were the two most influential pathological predictors of RFS (**Figure 2**). Notably, the PIIS demonstrated greater prognostic importance than any single hematological inflammatory marker.

### 3.4 Development and evaluation of the integrated nomogram

Univariable and multivariable Cox regression analyses identified sex, tumor size, mitotic index, Ki-67 index, and PIIS as independent prognostic factors for RFS (**Table 2**; **Supplementary Figure 12**). The relative importance of these variables was highly concordant with the SHAP results. Schoenfeld residual analyses showed no evidence of time-dependent effects, with both global and covariate-specific tests yielding P values > 0.05, confirming that the proportional hazards assumption was satisfied and that the estimated effects were stable (**Supplementary Figure 13**).

**Table 2:** Univariate and multivariate Cox regression analyses for recurrence-free survival (RFS) in the training cohort.

Factors	Univariate analysis		Multivariate analysis	
	HR (95%CI)	*P*-value	HR (95%CI)	*P*-value
Age (years)				
<60	1 (Reference)		1 (Reference)	
≥60	1.62 (1.05-2.50)	**0.029**	1.09 (0.66-1.81)	0.742
Sex				
Male	1 (Reference)		1 (Reference)	
Female	0.62 (0.40-0.95)	**0.029**	0.60 (0.37-0.98)	**0.043**
Tumor site				
Non-gastric gastrointestinal	1 (Reference)		1 (Reference)	
Extragastrointestinal	2.32 (1.41-3.81)	**<0.001**	1.36 (0.73-2.53)	0.336
Tumor size (cm)				
≤5	1 (Reference)		1 (Reference)	
>5 and ≤10	3.26 (1.65-6.44)	**<0.001**	3.20 (1.51-6.78)	**0.002**
>10	8.90 (4.69-16.91)	**<0.001**	2.78 (1.27-6.08)	**0.010**
Tumor necrosis				
No	1 (Reference)		1 (Reference)	
Yes	3.67 (2.37-5.69)	**<0.001**	1.19 (0.69-2.04)	0.530
Mitotic index (/50HPF)				
≤2	1 (Reference)		1 (Reference)	
>2 and ≤5	3.37 (1.78-6.37)	**<0.001**	2.66 (1.31-5.39)	**0.007**
>5 and ≤10	6.24 (3.12-12.47)	**<0.001**	3.30 (1.48-7.36)	**0.003**
>10	15.51 (7.71-31.22)	**<0.001**	4.16 (1.60-10.81)	**0.003**
Ki67 (%)				
≤5	1 (Reference)		1 (Reference)	
>5 and ≤10	5.98 (3.23-11.07)	**<0.001**	3.29 (1.58-6.86)	**0.001**
>10	10.52 (6.40-17.30)	**<0.001**	4.18 (2.18-8.03)	**<0.001**
Tumor rupture				
No	1 (Reference)		1 (Reference)	
Yes	2.95 (1.52-5.73)	**0.001**	0.74 (0.32-1.75)	0.497
Gene Mutation				
KIT exon 11	1 (Reference)		1 (Reference)	
Non-KIT exon 11	0.64 (0.28-1.44)	0.280	0.95 (0.40-2.26)	0.903
Not tested	0.53 (0.34-0.84)	**0.007**	1.30 (0.75-2.26)	0.353
PIIS	2.03 (1.38-3.00)	**<0.001**	2.52 (1.55 - 4.11)	**<0.001**

The bold values indicate statistically significant P-values (P<0.05).

**Figure 3:**
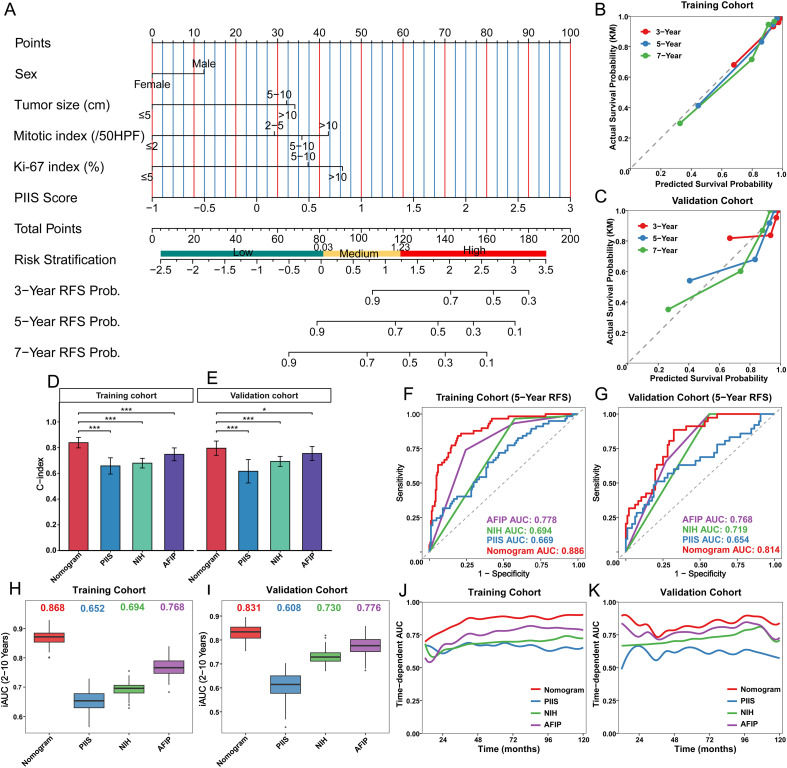
Development and validation of the integrated nomogram for recurrence-free survival (RFS). **(A)** The integrated nomogram for predicting 3-, 5-, and 7-year RFS. **(B, C)** Calibration curves for 3-, 5-, and 7-year RFS in the training **(B)** and validation **(C)** cohorts. **(D–G)** Comparison of C-index **(D, E)** and 5-year ROC curves **(F, G)** among the nomogram, PIIS, NIH, and AFIP systems. **(H–K)** iAUC boxplots **(H, I)** and time-dependent AUC curves **(J, K)** for the nomogram and comparison models. *P < 0.05; ***P < 0.001.

Based on these predictors, a nomogram was constructed to facilitate individualized estimation of 3-, 5-, and 7-year RFS probabilities (**Figure 3A**). Calibration curves demonstrated close agreement between predicted and observed outcomes in both the training and validation cohorts, indicating good calibration performance (**Figures 3B, C**).

In the training cohort, the nomogram achieved a C-index of 0.839 (95% CI, 0.798–0.879), which was significantly higher than that of the PIIS and the mNIH and AFIP criteria (all P < 0.001). This superior discrimination was consistently reproduced in the external validation cohort (C-index = 0.795) (**Figures 3D, E**; **Supplementary Table 1**). ROC analyses further confirmed that the nomogram outperformed comparator models in predicting 5-year RFS (**Figures 3F, G**). Consistently, iAUC and time-dependent ROC analyses showed that the nomogram maintained the highest and most stable AUC values over the 10-year follow-up period (**Figures 3H–K**). Moreover, the nomogram yielded the lowest AIC, BIC, and integrated Brier score among all models in both cohorts, indicating superior goodness-of-fit and predictive accuracy (**Supplementary Table 2**; **Supplementary Figures 14A, B**). DCA demonstrated a greater clinical net benefit across a wide range of threshold probabilities, underscoring its clinical utility (**Supplementary Figures 14C, D**). An interactive web-based calculator (https://gist.shinyapps.io/Gist-Rfs-Piis-Calculator/) derived from the nomogram has also been developed to support individualized RFS assessment in clinical practice (**Supplementary Figure 15**).

### 3.5 Prognostic stratification ability of the integrated nomogram

**Figure 4:**
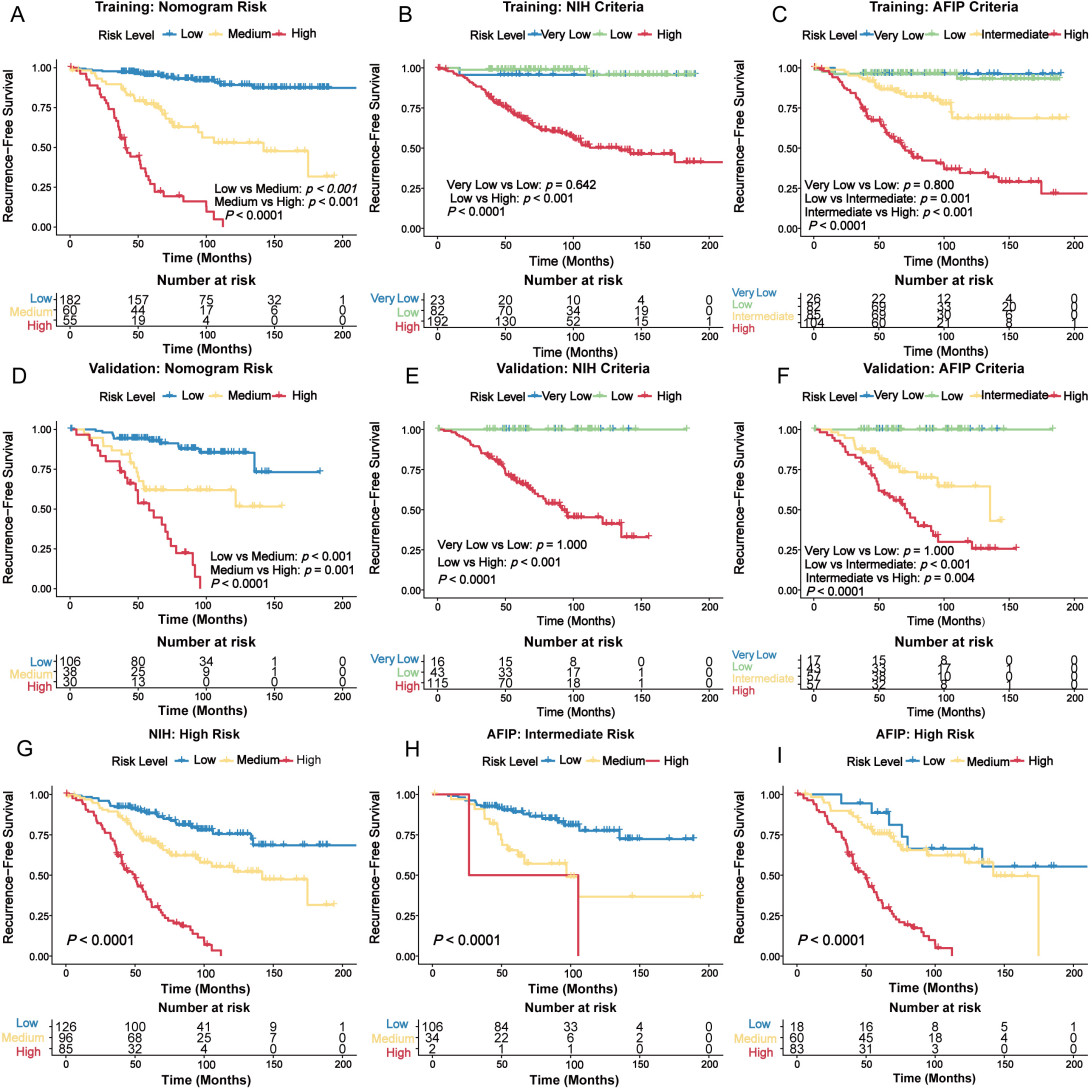
Comparison of risk stratification performance and clinical re-stratification capability. **(A–F)** Kaplan-Meier curves for recurrence-free survival (RFS) comparing the Nomogram risk categories with traditional modified NIH and AFIP criteria in the training cohort **(A–C)** and validation cohort **(D–F)**. **(G–I)** Re-stratification capability of the nomogram within specific traditional risk subgroups.

Using X-tile software, two optimal cutoff values of the total nomogram score were determined, stratifying patients into low-, medium-, and high-risk groups (**Supplementary Figure 16**). Kaplan–Meier analyses revealed clear limitations of conventional risk stratification systems. Under the mNIH criteria, an intermediate-risk category could not be clearly delineated, and substantial overlap was observed between the “very low-risk” and “low-risk” groups, with no significant survival difference (P > 0.05), indicating limited discriminatory capacity within the low-risk spectrum. Similarly, the AFIP classification failed to achieve effective prognostic separation between its “very low-risk” and “low-risk” categories (P > 0.05). In contrast, the integrated nomogram demonstrated markedly superior stratification performance. It effectively distinguished patients across low-, medium-, and high-risk categories, with statistically significant survival differences between all adjacent groups. Moreover, the P values for adjacent-group comparisons were consistently smaller than those obtained using the mNIH and AFIP criteria, underscoring the enhanced discriminatory resolution of the nomogram (**Figures 4A–F**).

To further assess its value in clinically high-risk populations, re-stratification analyses were performed among patients classified as “high risk” by the mNIH criteria and those categorized as “intermediate/high risk” by the AFIP system. The nomogram successfully identified prognostically distinct subgroups within these traditionally high-recurrence populations (all P < 0.0001), revealing substantial intragroup heterogeneity (**Figures 4G–I**). Sankey diagrams further illustrated dynamic patient migration across different risk systems, demonstrating that the nomogram refined the coarse stratification of the NIH and AFIP criteria by reallocating a subset of patients originally labeled as “high risk” into lower-risk categories, with improved concordance to actual recurrence outcomes (**Supplementary Figure 17**).

**Figure 5:**
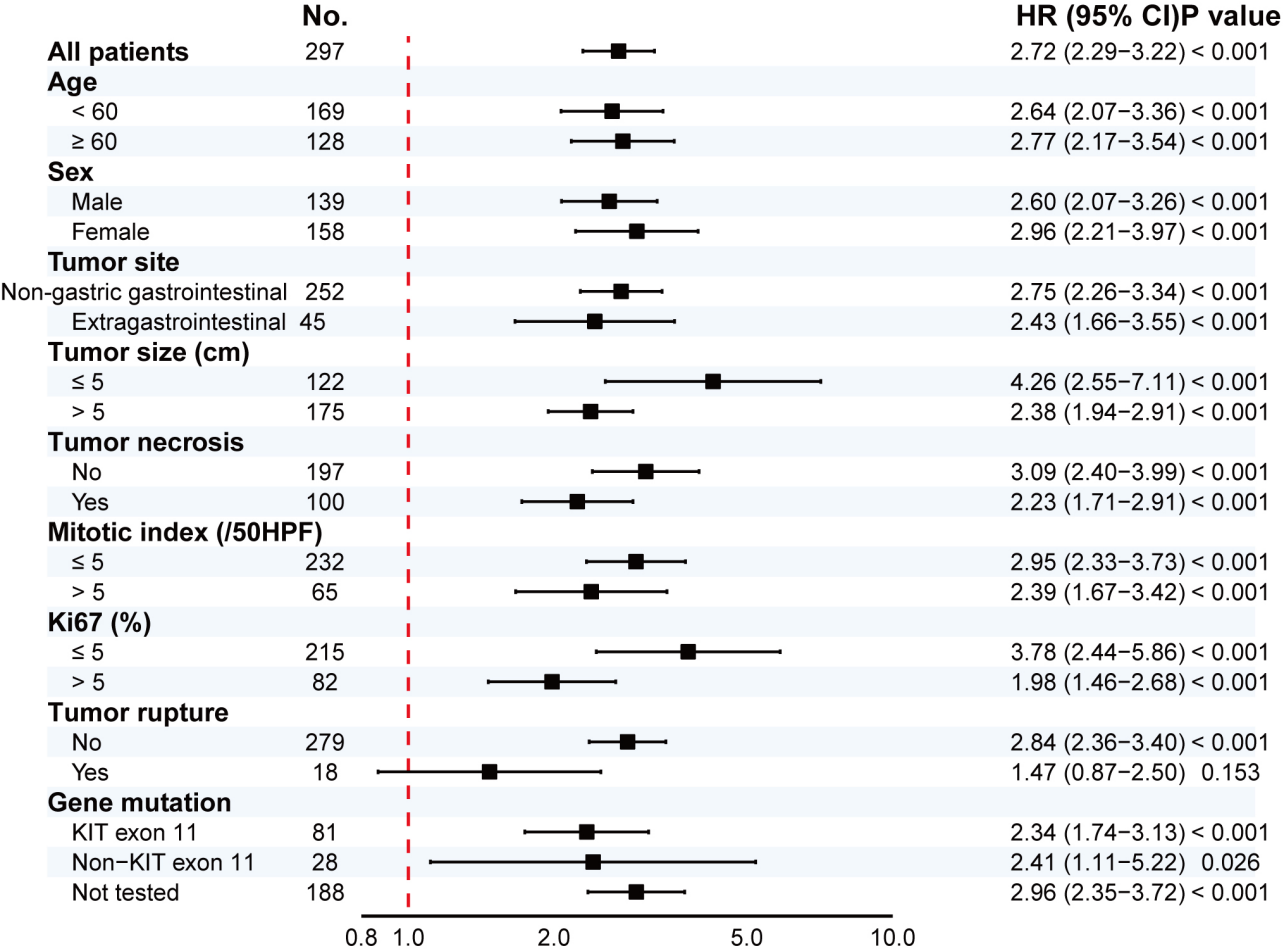
Subgroup analysis of recurrence-free survival (RFS) using nomogram score in the training cohort. Black squares and horizontal lines represent the estimated hazard ratios (HRs) and their corresponding 95% CIs, respectively.

The generalizability of the nomogram was further evaluated across clinical subgroups. Forest plots from the training cohort showed that the nomogram-based risk score remained a significant predictor of RFS in nearly all subgroups, except for patients with tumor rupture (**Figure 5**). These findings were consistently reproduced in the external validation cohort (**Supplementary Figure 18**). Subgroup-specific Kaplan–Meier analyses further confirmed clear risk separation among nomogram-defined groups within each subgroup (**Supplementary Figure 19**). Although statistical significance was not reached in the tumor rupture subgroup due to limited sample size, the direction and magnitude of the effect were consistent with those observed in the overall cohort.

### 3.6 Heatmap analysis of nomogram variables

**Figure 6:**
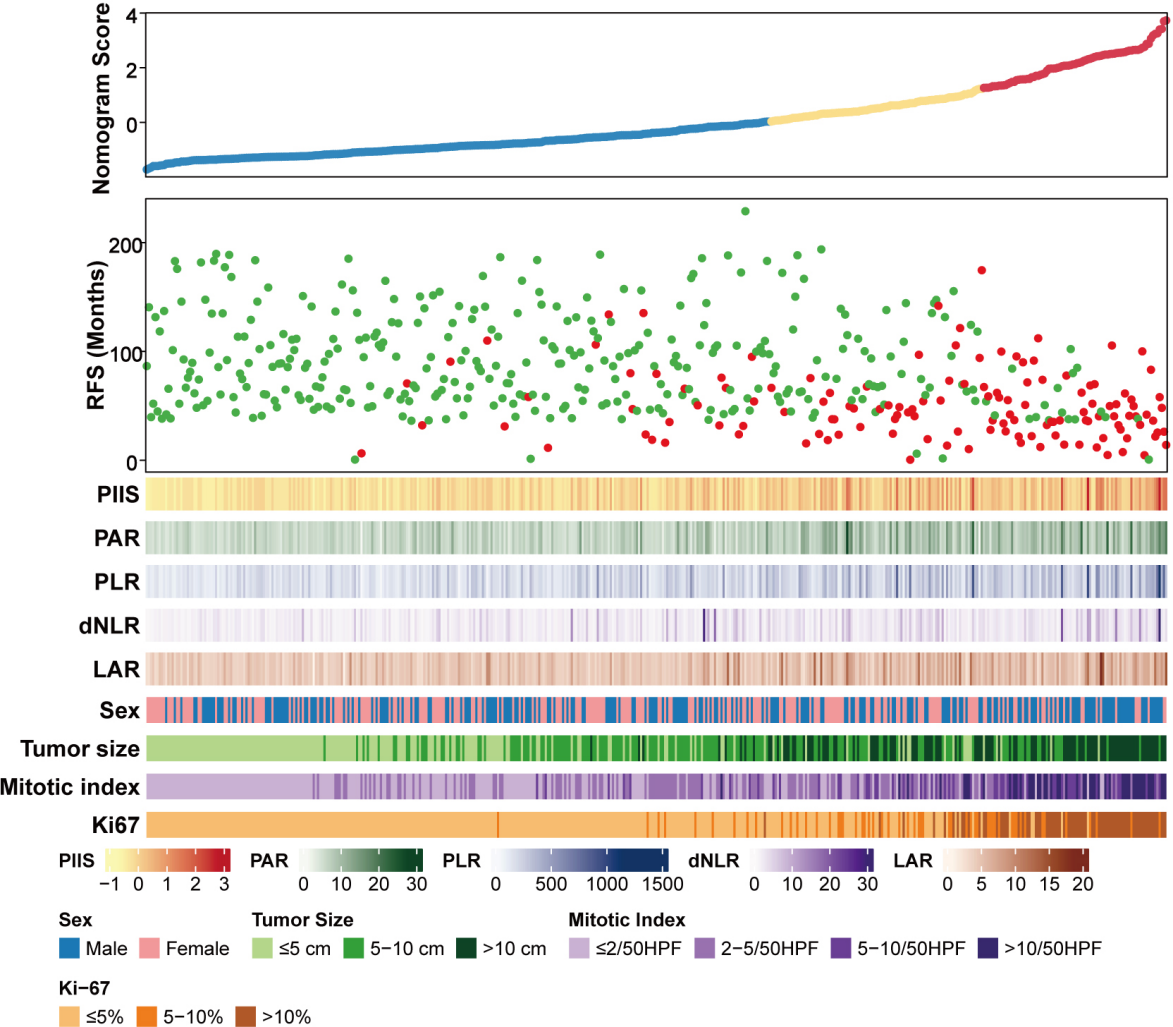
Distribution of risk scores and clinicopathological features in the overall cohort. Top: Patients ranked by ascending nomogram scores, colored by risk category (Low, Medium, and High). Middle: Corresponding recurrence status and follow-up duration, with red and green dots representing events and censored cases, respectively. Bottom: Heatmap illustrating the distribution of individual prognostic features for each patient.

To visualize multidimensional associations among nomogram-predicted risk, clinicopathological features, and survival outcomes, risk heatmaps were generated for the entire cohort (**Figure 6**), the training cohort (**Supplementary Figure 20**), and the external validation cohort (**Supplementary Figure 21**). Patients were ordered by increasing nomogram score, with color gradients illustrating the distribution of individual predictors alongside RFS time and event status. The heatmaps revealed a clear and consistent risk gradient. With increasing nomogram scores, recurrence events became progressively more frequent and RFS duration markedly shortened. High-risk regions showed pronounced enrichment of adverse prognostic features, including larger tumor size, higher mitotic index, elevated Ki-67, and increased PIIS. These patterns were highly consistent across the training and validation cohorts, supporting the nomogram’s strong discriminatory ability and robust generalizability across populations.

### 3.7 Risk-based benefit of prolonged adjuvant imatinib therapy

**Figure 7:**
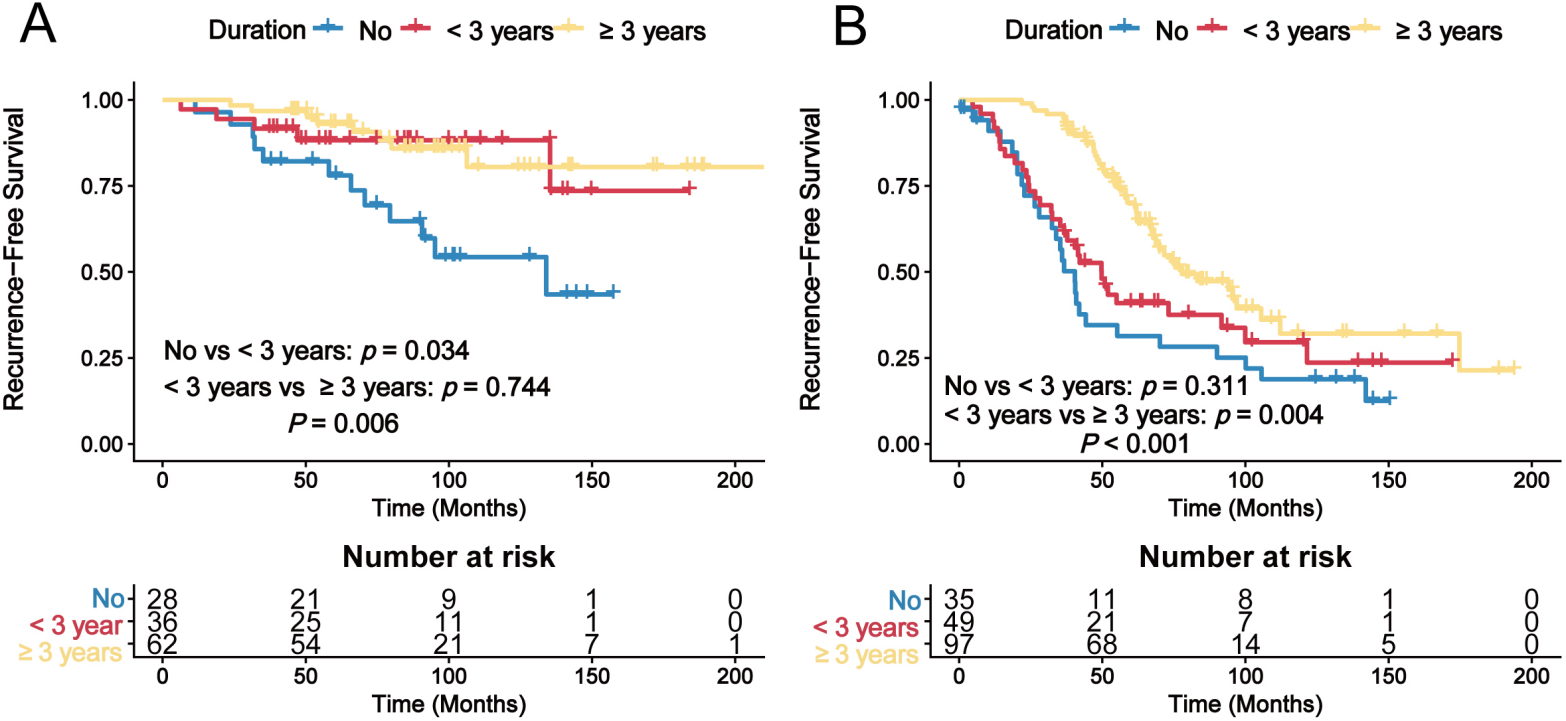
Recurrence-free survival (RFS) benefits of prolonged imatinib therapy in modified NIH high-risk patients stratified by the nomogram. Kaplan–Meier RFS curves were plotted and compared based on imatinib treatment duration for the **(A)** low-risk and **(B)** medium/high-risk subgroups.

Among patients classified as high risk by the mNIH criteria—who are clinically recommended to receive 3 years of adjuvant therapy—exploratory stratified analyses demonstrated that the nomogram effectively identified subgroups with differential benefit from treatment duration. In patients reclassified as low risk by the nomogram (**Figure 7A**), adjuvant therapy was associated with significantly improved survival compared with no treatment; however, extending therapy beyond 3 years did not confer additional benefit relative to treatment for less than 3 years (P = 0.744), indicating limited value of prolonged therapy in this subgroup. In contrast, patients classified as medium and high risk by the nomogram (**Figure 7B**) exhibited a clear duration-dependent treatment effect. While short-term therapy (<3 years) showed a nonsignificant trend toward improved survival compared with no treatment (P = 0.311), only treatment durations exceeding 3 years resulted in a significantly superior survival outcome (P = 0.004). These findings suggest that survival improvement in this nomogram-defined “true high-risk” population may be associated with adequate duration of adjuvant imatinib therapy.

## 4 Discussion

This multicenter cohort–based study focused on non-gastric GISTs, a subgroup characterized by pronounced prognostic heterogeneity, and developed and validated an integrated predictive system that combines host immuno-inflammatory features with classical pathological parameters. Through a rigorous machine learning–driven feature selection strategy, we established the PIIS as an independent prognostic factor in non-gastric GIST. More importantly, the proposed model not only outperformed conventional risk stratification systems, including the mNIH and AFIP criteria, in terms of predictive accuracy, but also successfully uncovered substantial prognostic heterogeneity within patients traditionally classified as high risk. This capability highlights its translational value in guiding the optimal duration of adjuvant imatinib therapy. Collectively, these findings may provide a useful reference for optimizing the postoperative management of non-gastric GIST, helping to avoid both overtreatment and undertreatment.

Before discussing the clinical implications of our findings, it is necessary to critically examine the limitations of existing risk stratification systems for non-gastric GIST. For decades, the mNIH and AFIP classifications have guided clinical decision-making; however, both are inherently affected by a pronounced “anatomical bias.” As approximately 60% of GISTs arise in the stomach, these criteria were largely derived from gastric-dominant cohorts, limiting their applicability to non-gastric tumors (7, 8). Biologically, non-gastric GISTs exhibit more aggressive behavior and higher early recurrence rates, even in tumors with small size and low mitotic index, which may still be associated with unfavorable outcomes (23, 24). Our study clearly reveals this “assessment blind spot.” In the training cohort, survival curves for the very low– and low-risk groups defined by either mNIH or AFIP criteria were nearly overlapping. Notably, under the mNIH system, no non-gastric GIST patients met the intermediate-risk criteria, resulting in a complete absence of this stratum and substantially limiting refined surveillance in clinical practice. Moreover, traditional systems rely on discretized cutoffs (e.g., tumor size of 5 cm or mitotic index of 5 per 50 HPFs), adopting a stepwise model that ignores the continuous nature of biological variables. Consequently, cases near threshold values are highly susceptible to risk misclassification. For instance, under mNIH criteria, a 1-mm increase in tumor size (from 5.0 to 5.1 cm) results in abrupt reclassification from low to high risk (7), while AFIP data show that recurrence risk in small intestinal GIST increases sharply from 4.3% to 24% (8). Such discontinuous “risk cliffs” caused by trivial measurement differences underscore the biological limitations of conventional systems and may directly influence the decision to initiate adjuvant imatinib therapy.

To address these limitations, we introduced the PIIS, an integrated score incorporating multiple peripheral blood–based biomarkers. Although tumor progression is fundamentally driven by genomic alterations, accumulating evidence indicates that the host’s systemic inflammatory–immune status plays a pivotal role in shaping the tumor microenvironment and in determining the fate of minimal residual disease (MRD) (25, 26). By integrating four indices—PAR, PLR, dNLR, and LAR—PIIS effectively constructs a multidimensional profile of host inflammatory–immune status. LDH levels reflect underlying tumor burden and metabolic activity, whereas dynamic alterations in NEU, PLT, and LYM ratios characterize the strength of host antitumor immune surveillance (27–30). Accordingly, a high PIIS may represent a host milieu dominated by pro-tumorigenic inflammation, immune evasion, and metabolic reprogramming, providing a plausible biological explanation for its strong association with increased recurrence risk. Our analyses confirmed that PIIS is not only an independent prognostic factor but also demonstrates remarkable robustness across all subgroup analyses. Notably, SHAP-based interpretability analysis revealed that PIIS contributed more to recurrence risk prediction than any single hematological parameter, underscoring the superiority of multi-indicator integration in mitigating individual physiological fluctuations and enhancing predictive stability. The visualized nomogram developed in this study synergistically integrates key clinicopathological variables with PIIS, achieving an optimal balance between predictive accuracy and clinical applicability. Through this “dimensionally complementary” strategy, the model enables bidirectional risk discrimination: it identifies patients classified as low risk by conventional criteria but harboring unfavorable immuno-inflammatory states, as well as patients deemed high risk by traditional systems but exhibiting favorable host immune profiles, thereby substantially refining clinical risk stratification.

Precisely reconstructing intragroup heterogeneity within traditionally high–recurrence-risk populations represents a major clinical contribution of this study. Under current guidelines, non-gastric GIST patients classified as intermediate or high risk by the mNIH or AFIP criteria are generally managed as a homogeneous group and receive similar therapeutic strategies. In contrast, the nomogram developed in this study effectively dissects latent prognostic heterogeneity within this population, enabling a second-level refinement beyond conventional stratification. Sankey diagram–based migration analyses intuitively demonstrate how the model reallocates a subset of patients misclassified as high risk by traditional systems into intermediate- or low-risk categories. Importantly, the model showed strong clinical generalizability. Subgroup analyses consistently demonstrated significant prognostic discrimination across diverse clinicopathological strata. This cross-subgroup stability indicates that the system is not driven by any single variable, but instead captures shared biological trajectories of non-gastric GIST progression through multidimensional calibration.

The benefit of adjuvant therapy after resection of non-gastric GIST is not uniform, but instead exhibits marked interindividual variability. Although the SSG XVIII/AIO trial demonstrated that 3 years of adjuvant imatinib improves RFS and overall survival (OS) in high-risk patients (31), the pronounced surge in recurrence following treatment discontinuation suggests that imatinib primarily maintains highly aggressive clones in a state of “drug-induced dormancy” through kinase inhibition, rather than achieving complete eradication (32). Within the medium- and high-risk groups identified by our nomogram, we similarly observed a characteristic post-discontinuation recurrence spike, closely mirroring the core findings of the IMADGIST trial—namely, that patients at extremely high risk exhibit strong rebound potential after withdrawal of pharmacologic pressure, and that prolonged adjuvant therapy significantly improves outcomes in this subgroup (33). Importantly, the model enabled precise benefit stratification. Among patients reclassified as low risk, extended treatment conferred no additional survival benefit (P = 0.744), indicating relatively indolent tumor biology for which standard-duration therapy is sufficient. In contrast, patients in the intermediate- and high-risk subgroups demonstrated a clear time-dependent survival advantage, with significant benefit observed only when adjuvant therapy was extended beyond 3 years (P = 0.004). These exploratory findings suggest that nomogram-defined risk strata may help identify patients who are more likely to derive benefit from prolonged adjuvant imatinib therapy, although prospective validation is required.

To our knowledge, this is the first study to systematically apply machine learning to screen and integrate multiple peripheral blood–derived inflammatory–immune markers into a composite score (PIIS) and to develop a multicenter-validated, individualized prognostic model specifically for non-gastric GIST. However, several limitations should be noted. First, the retrospective design may introduce inherent bias, and the model’s robustness and generalizability require confirmation in prospective cohorts. Second, overall survival (OS)-based analysis was not performed because death events were relatively infrequent in this cohort, with deaths observed in only 12.9% of patients. Moreover, OS in GIST may be affected by post-recurrence imatinib therapy, subsequent tyrosine kinase inhibitor treatment, and non-tumor-related mortality, thereby limiting the reliability of OS-based model construction. Third, the retrospective comparison of adjuvant treatment duration may be affected by treatment indication bias and time-dependent bias and should therefore be interpreted as exploratory. Fourth, hematological variables included in the PIIS may be influenced by transient conditions such as infection or anemia; dynamic assessment or averaging across multiple time points may enhance stability and reproducibility. Fifth, the high proportion of missing gene mutation data in our cohort may introduce potential bias and limit the statistical power of subgroup analyses stratified by mutation status; therefore, extrapolating these findings to specific molecular subgroups requires caution. Furthermore, because only treatment-naïve patients without neoadjuvant therapy were included, the model’s predictive performance in patients receiving neoadjuvant treatment remains to be determined. Finally, although a web-based calculator was developed to enhance practical utility, as an adjunctive tool derived from retrospective data, its predictions should serve merely as a reference for clinical decision-making and should not replace the comprehensive clinical judgment of the attending physician based on the patient’s overall condition.

## 5 Conclusion

This study developed and validated a prognostic prediction model specifically tailored for non-gastric GIST. By integrating PIIS, a composite indicator of host systemic inflammatory–immune status, with established pathological features, this model may help overcome key limitations of mNIH and AFIP classifications and enable more precise and continuous risk stratification. Importantly, it identifies patients who are most likely to benefit from prolonged adjuvant imatinib therapy. Overall, this model may provide a useful reference for individualized prognostic evaluation and precise adjuvant therapy decision-making in non-gastric GIST, although its clinical applicability requires further validation in prospective studies.

## Data Availability

The raw data supporting the conclusions of this article will be made available by the authors, without undue reservation.
