# Supplementary material for: Prognostic inflammatory–immune score-based risk stratification optimizes adjuvant therapy for non-gastric gastrointestinal stromal tumors: a multicenter study
Source: Front Immunol. 2026 Jun 2;17:1846854. doi: 10.3389/fimmu.2026.1846854 (PMC13269074; doi:10.3389/fimmu.2026.1846854)
Supplement: Supplementary file 1 [file Table1.docx]

**Supplementary Figures**

**
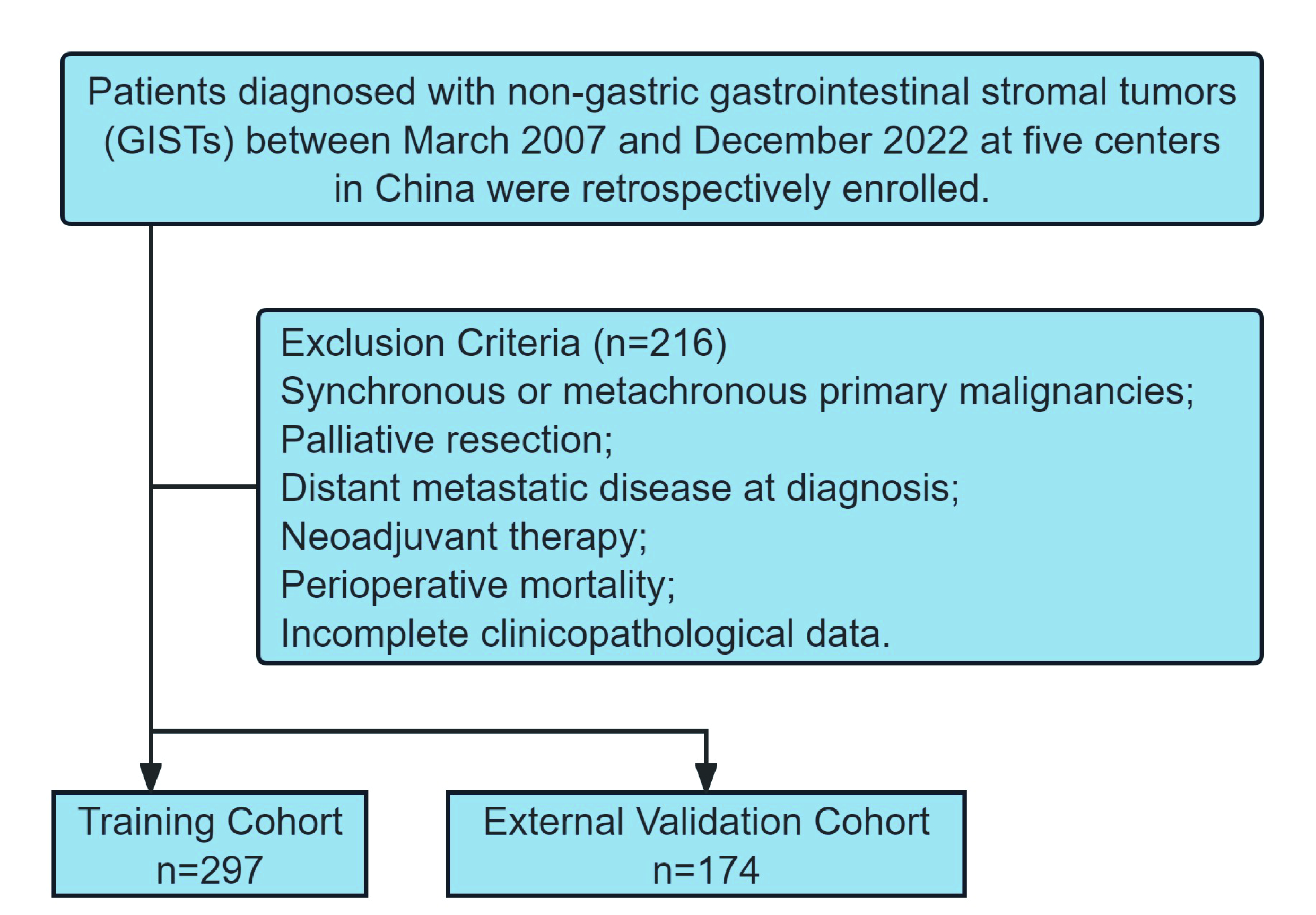
**

**Figure S1. Recruitment pathways for patients in the training and external validation cohorts.**

**
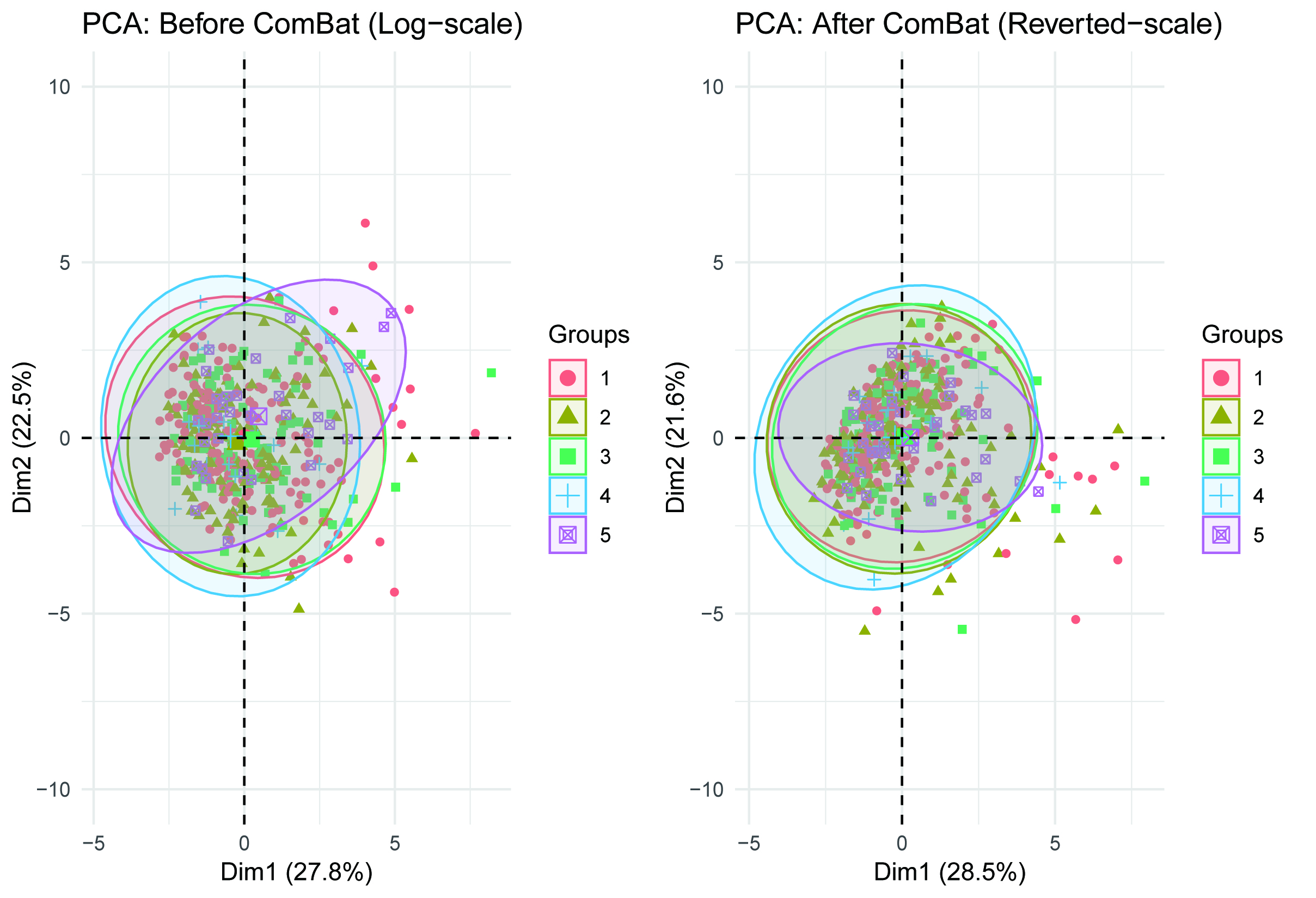
**

**Figure S2. Principal component analysis (PCA) for batch effect correction.** (A) PCA plot of log-transformed hematological data before ComBat harmonization. (B) PCA plot after ComBat harmonization. Groups 1–5 represent the five participating medical centers.

**
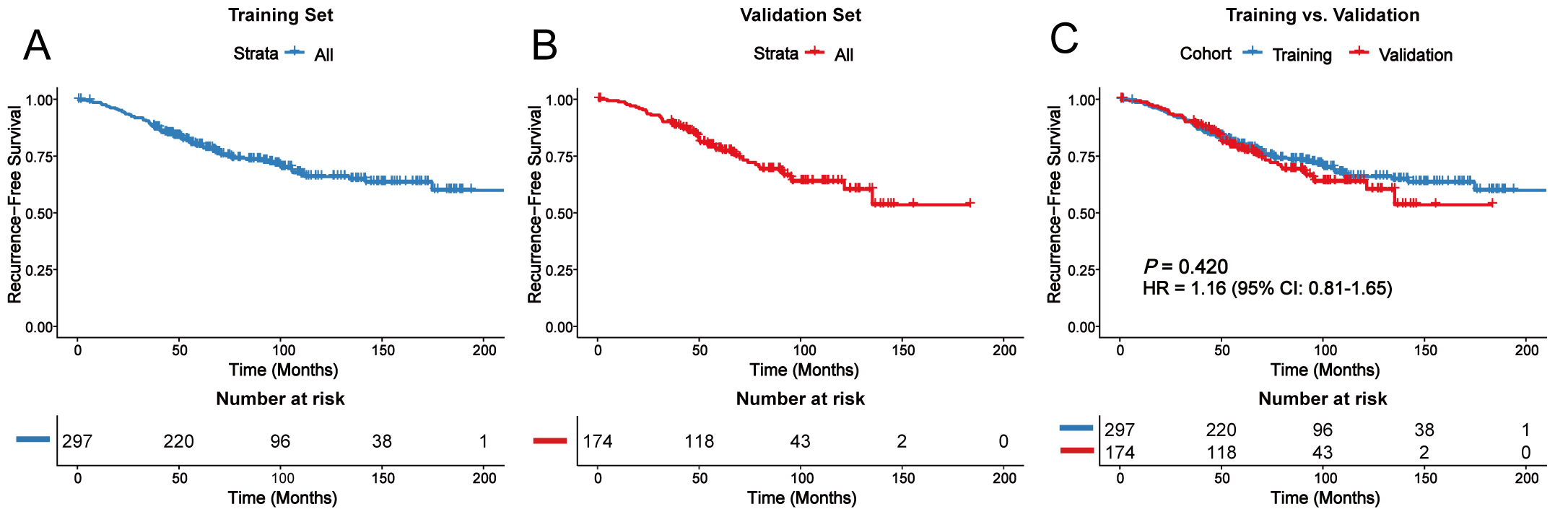
**

**Figure S3. Kaplan-Meier survival analysis in the training and validation cohorts.** (A) Recurrence-free survival (RFS) curve in the training cohort. (B) RFS curve in the validation cohort. (C) Evaluation of the survival differences between the two cohorts.

**
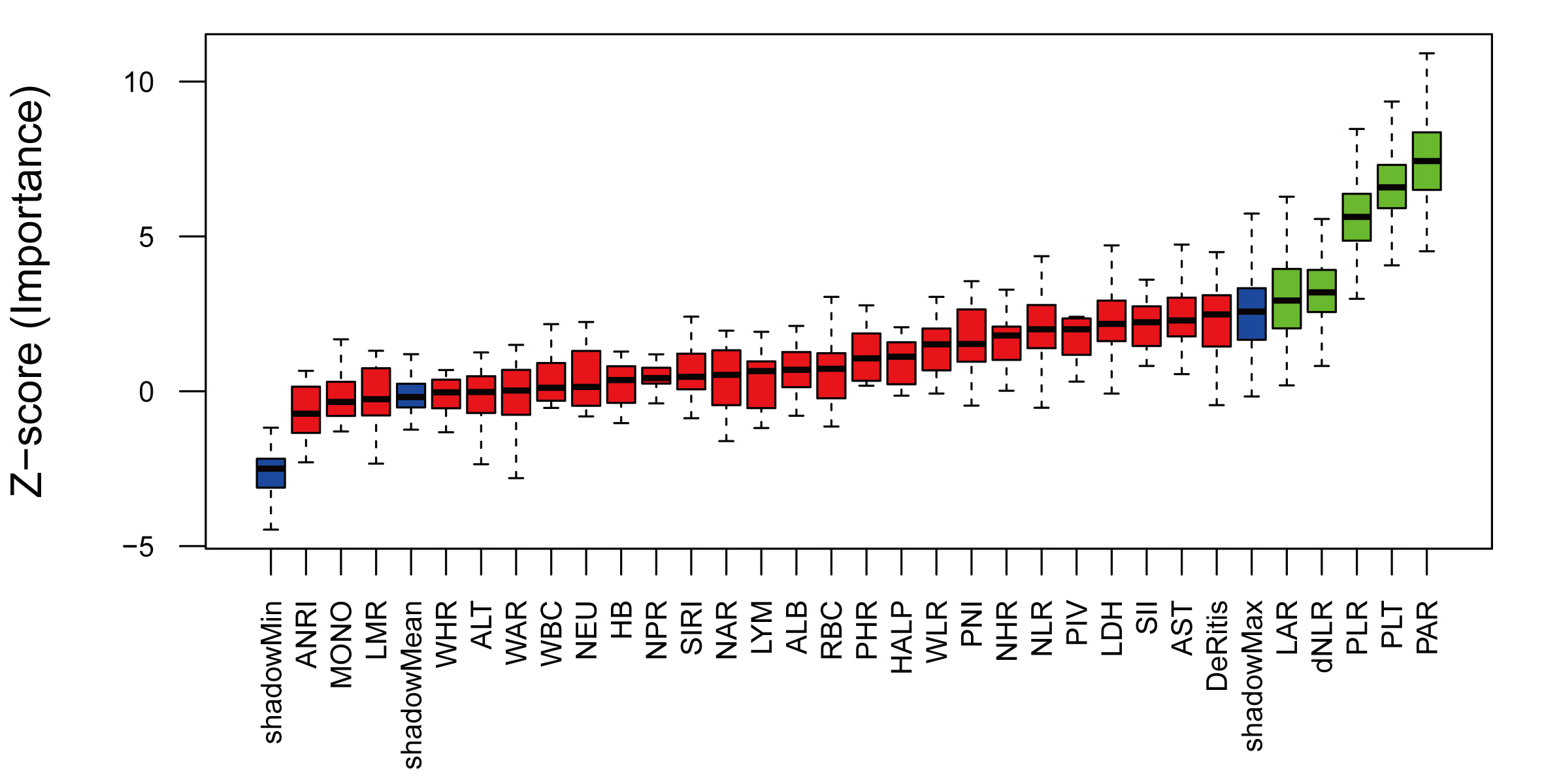
**

**Figure S4. Feature selection and importance ranking based on the Boruta algorithm.** Green boxplots indicate confirmed important features with higher Z-scores than shadow features (blue), whereas red boxplots represent rejected variables.

**
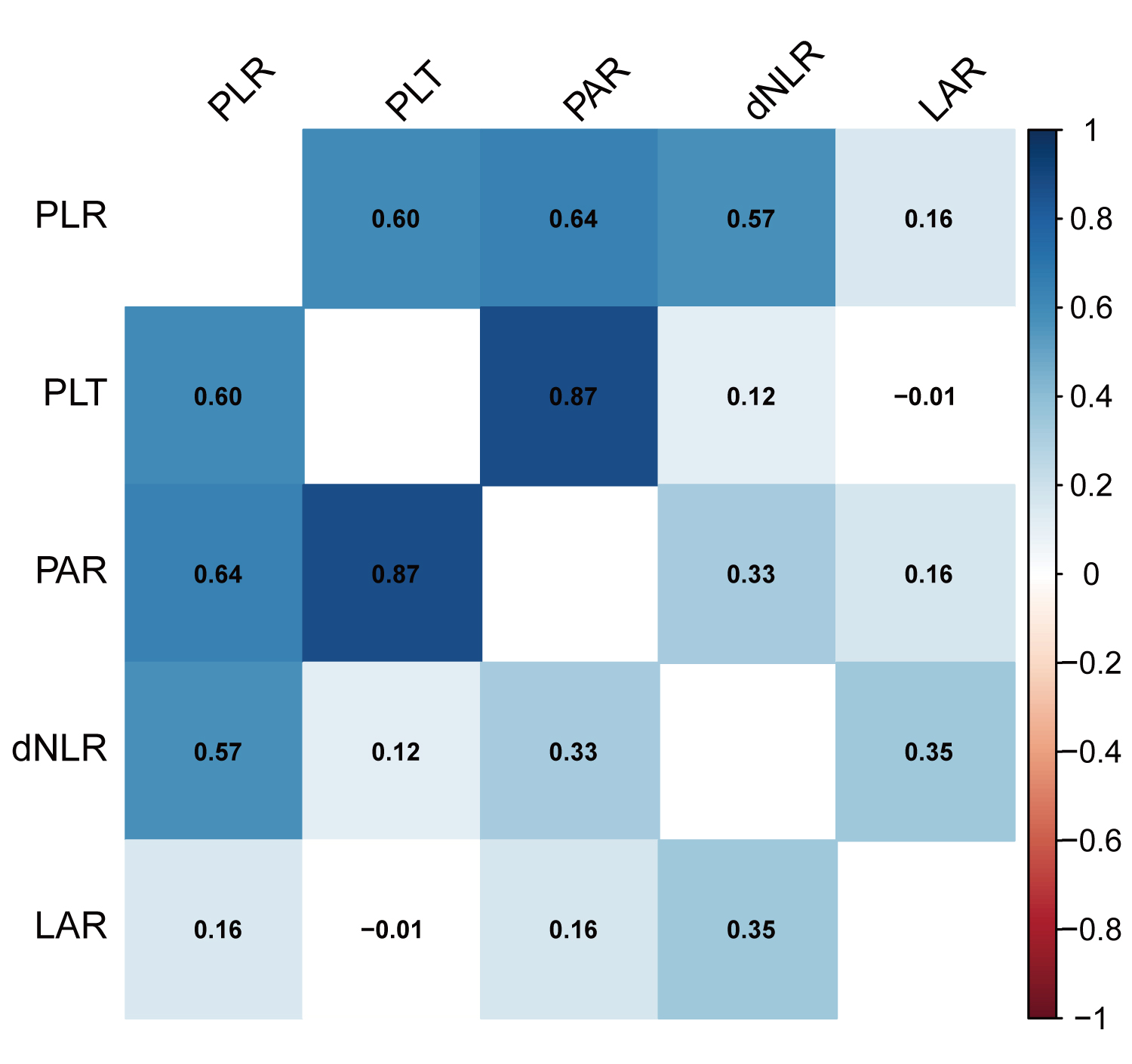
**

**Figure S5. Correlation matrix of Boruta-selected features.** Spearman correlation coefficients among the five identified features. Color intensity (blue) represents the strength of positive correlation.


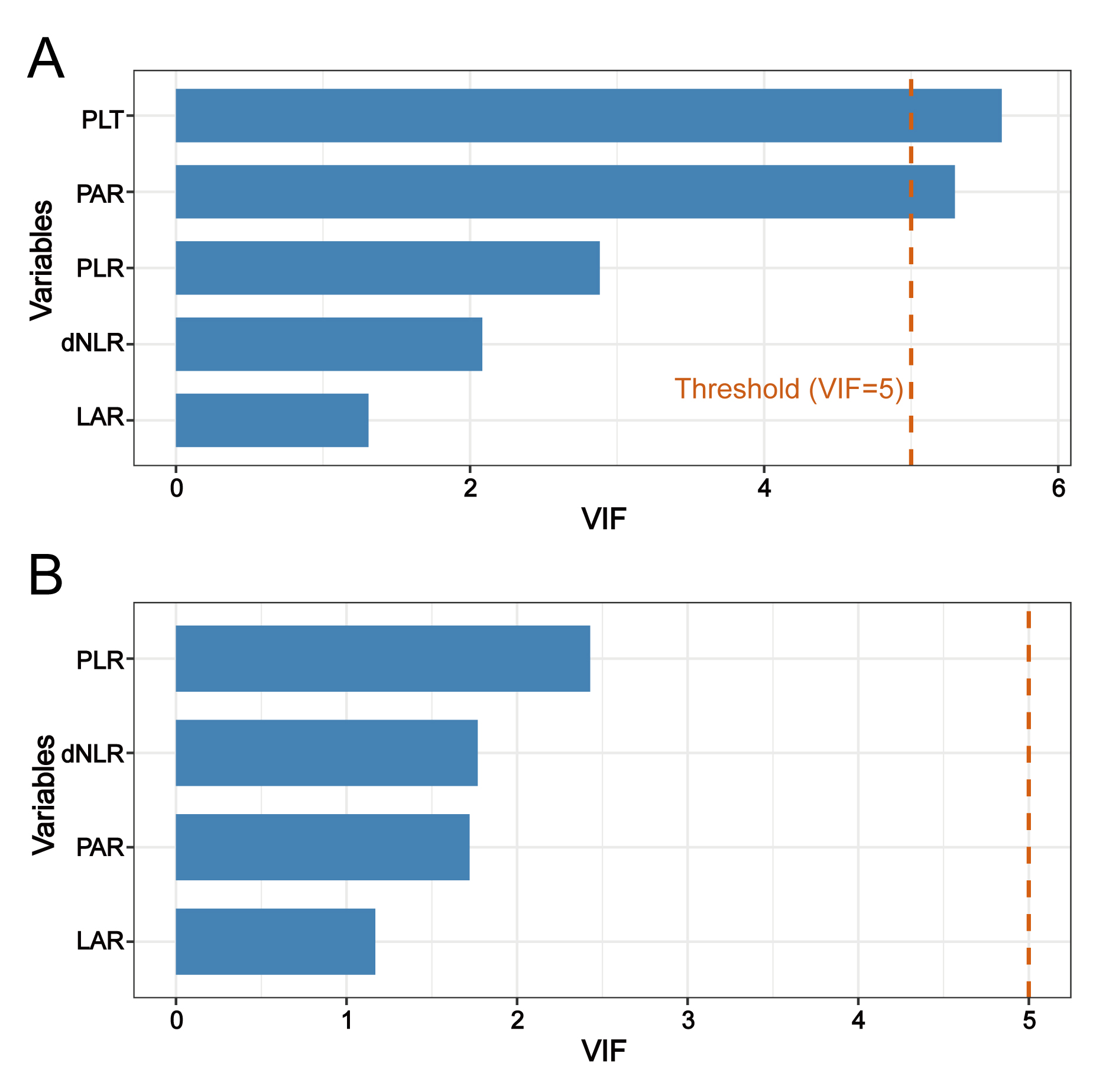


**Figure S6. Multicollinearity screening via variance inflation factor (VIF).** (A) Initial VIF values including PLT and PAR. (B) Final VIF values for the four features (PLR, dNLR, PAR, LAR) after iterative elimination of PLT. Red dashed line indicates the VIF threshold of 5.


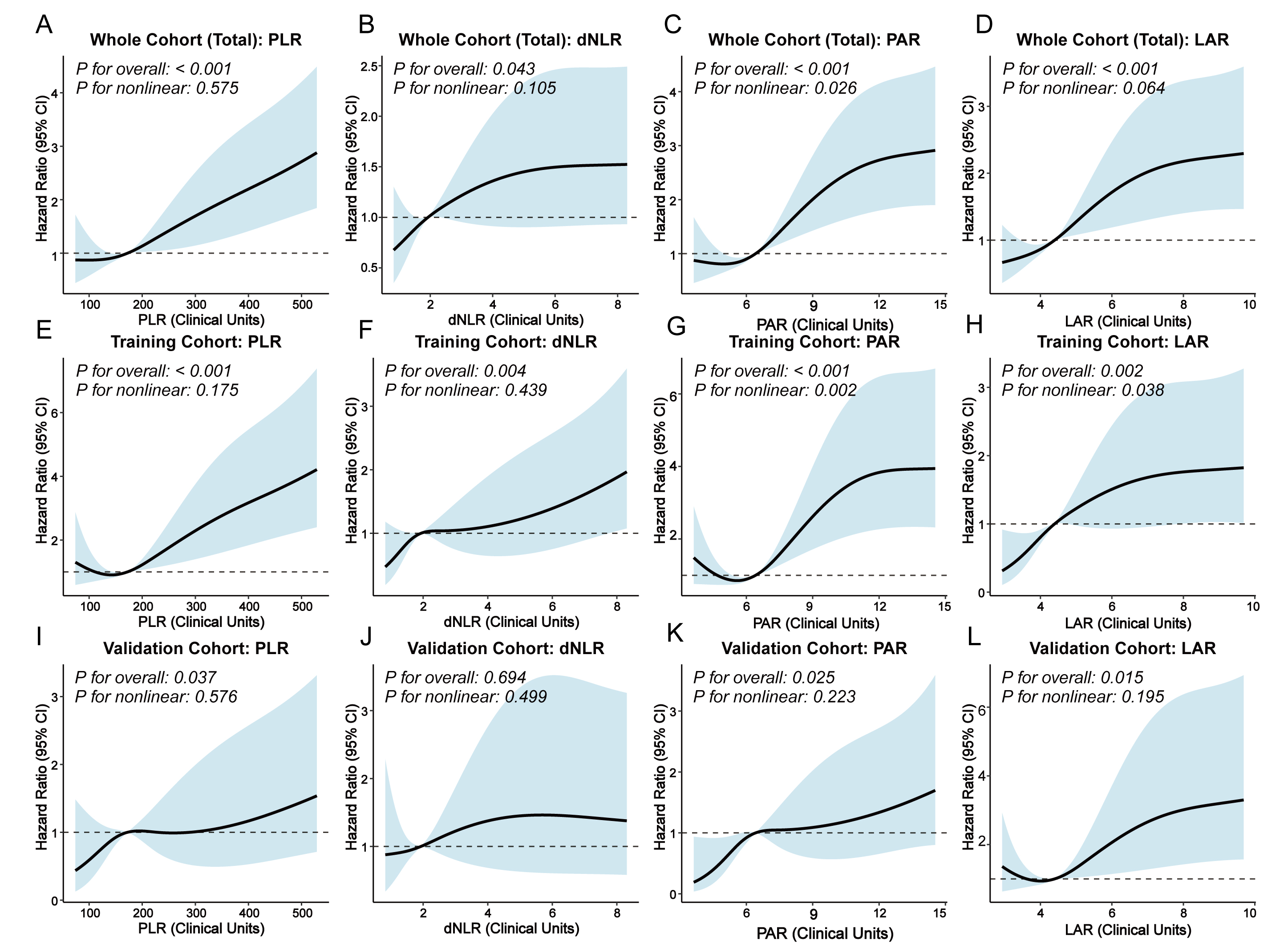


**Figure S7 Restricted cubic spline analysis for the dose-response association between systemic inflammatory markers and RFS.** PLR, dNLR, PAR and LAR were analyzed in the overall (A–D), training (E–H) and validation (I–L) cohorts. Solid black lines represent hazard ratios, and shaded regions indicate 95% confidence intervals. Horizontal dashed lines denote the reference HR of 1.0.

**
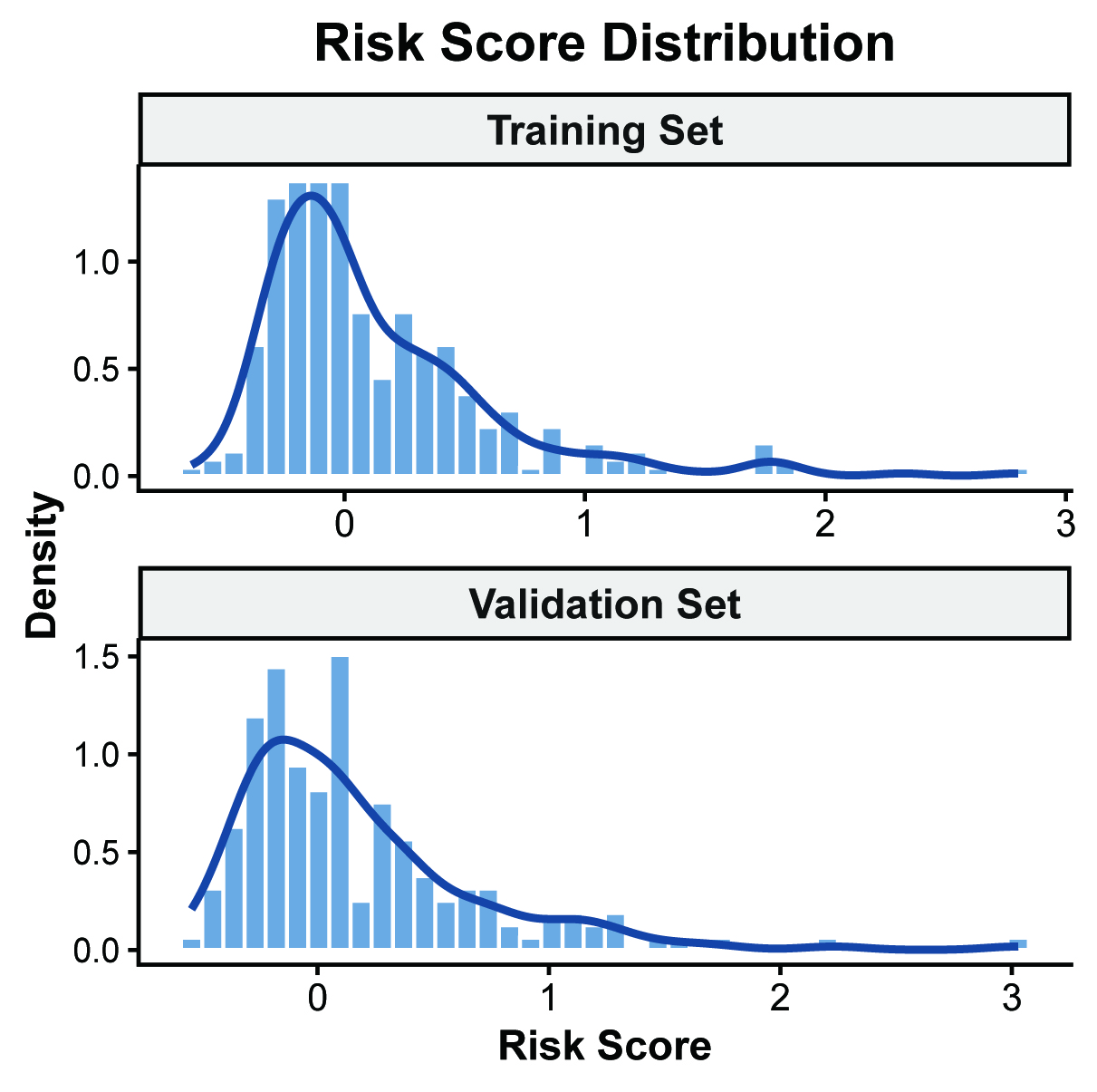
**

**Figure S8. Distribution histogram of PIIS-score in the training and external validation cohorts.**

**
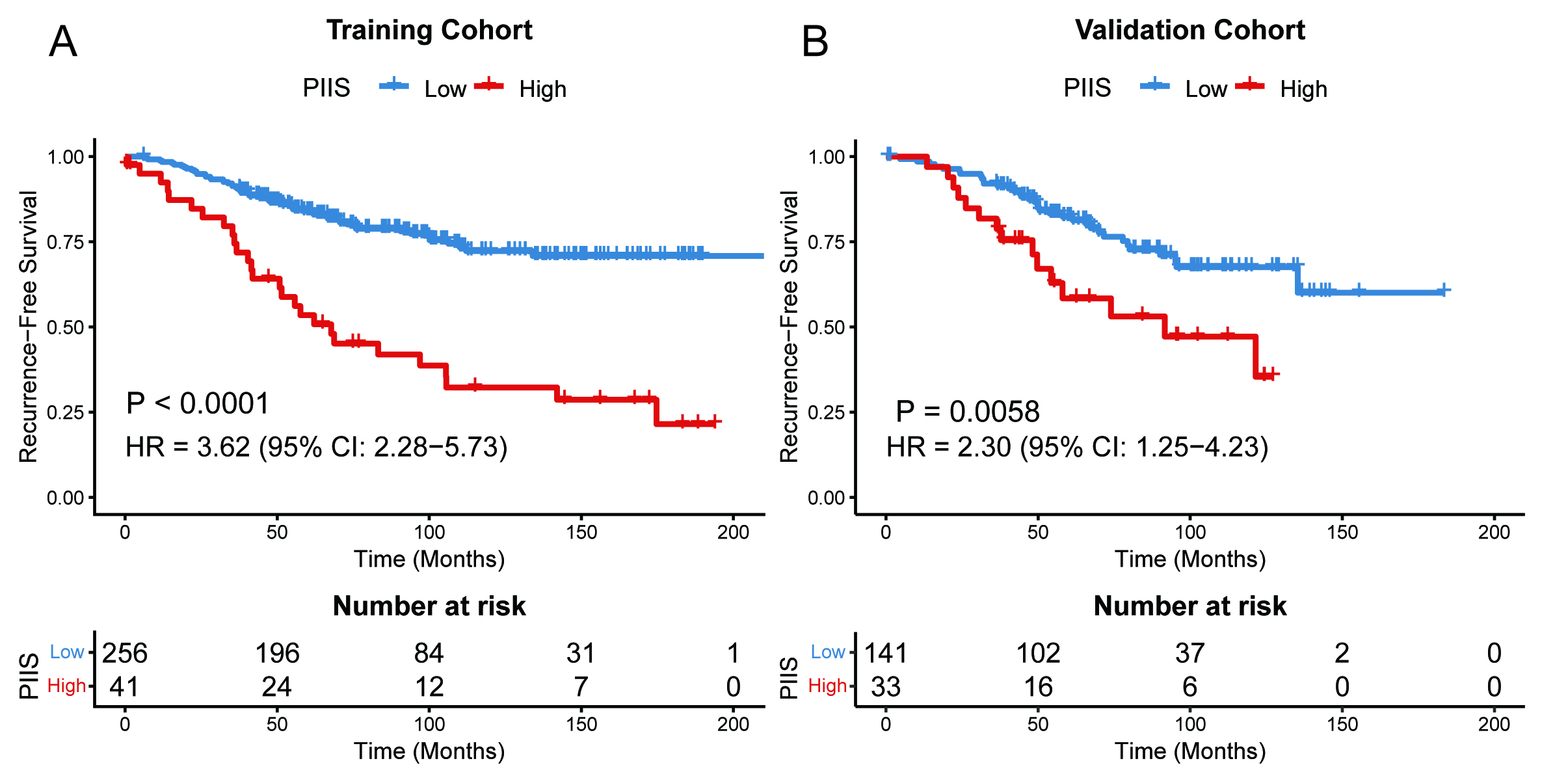
**

**Figure S9. Recurrence-free survival (RFS) stratified by PIIS categories.** Kaplan–Meier curves comparing high- and low-PIIS groups in the (A) training cohort and (B) validation cohort.

**
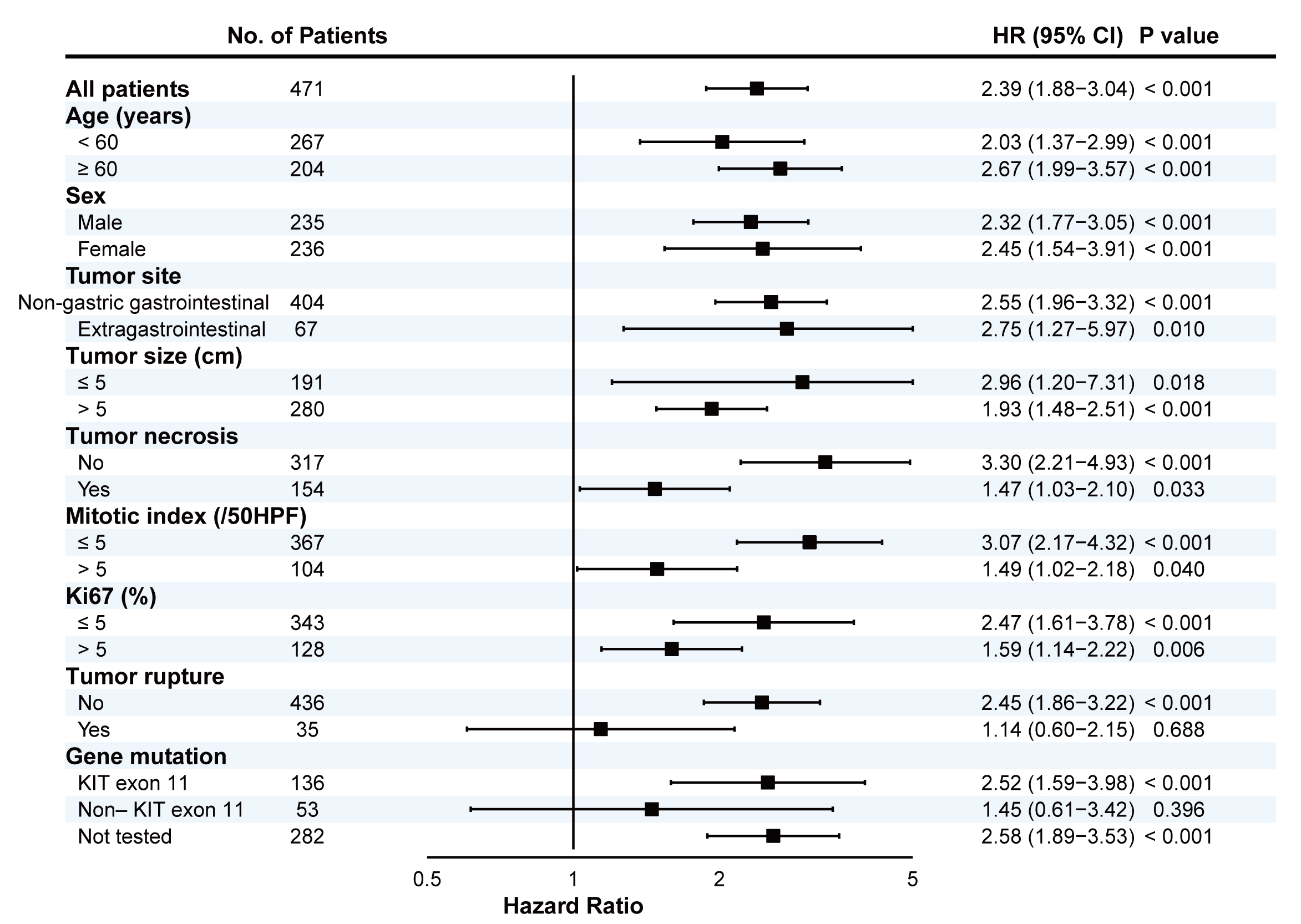
Figure S10. Subgroup analysis of recurrence-free survival (RFS) according to the PIIS-score.** Black squares and horizontal lines represent the estimated HRs and their corresponding 95% CIs, respectively.

**
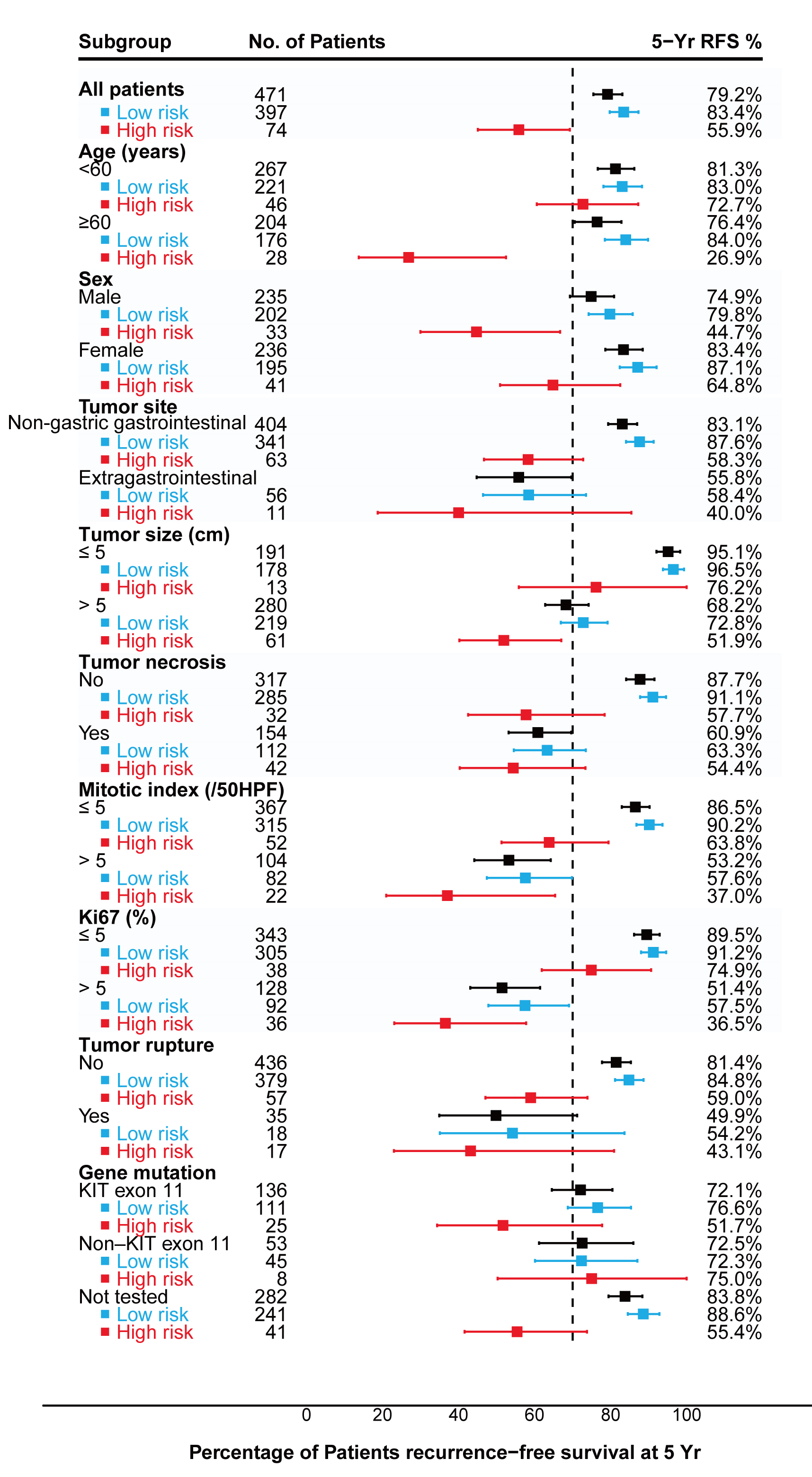
**

**Figure S11. Five-year recurrence-free survival (RFS) rates stratified by the PIIS-score across subgroups.** For each subgroup, risk categories were defined by the optimal PIIS-score cutoff.

**
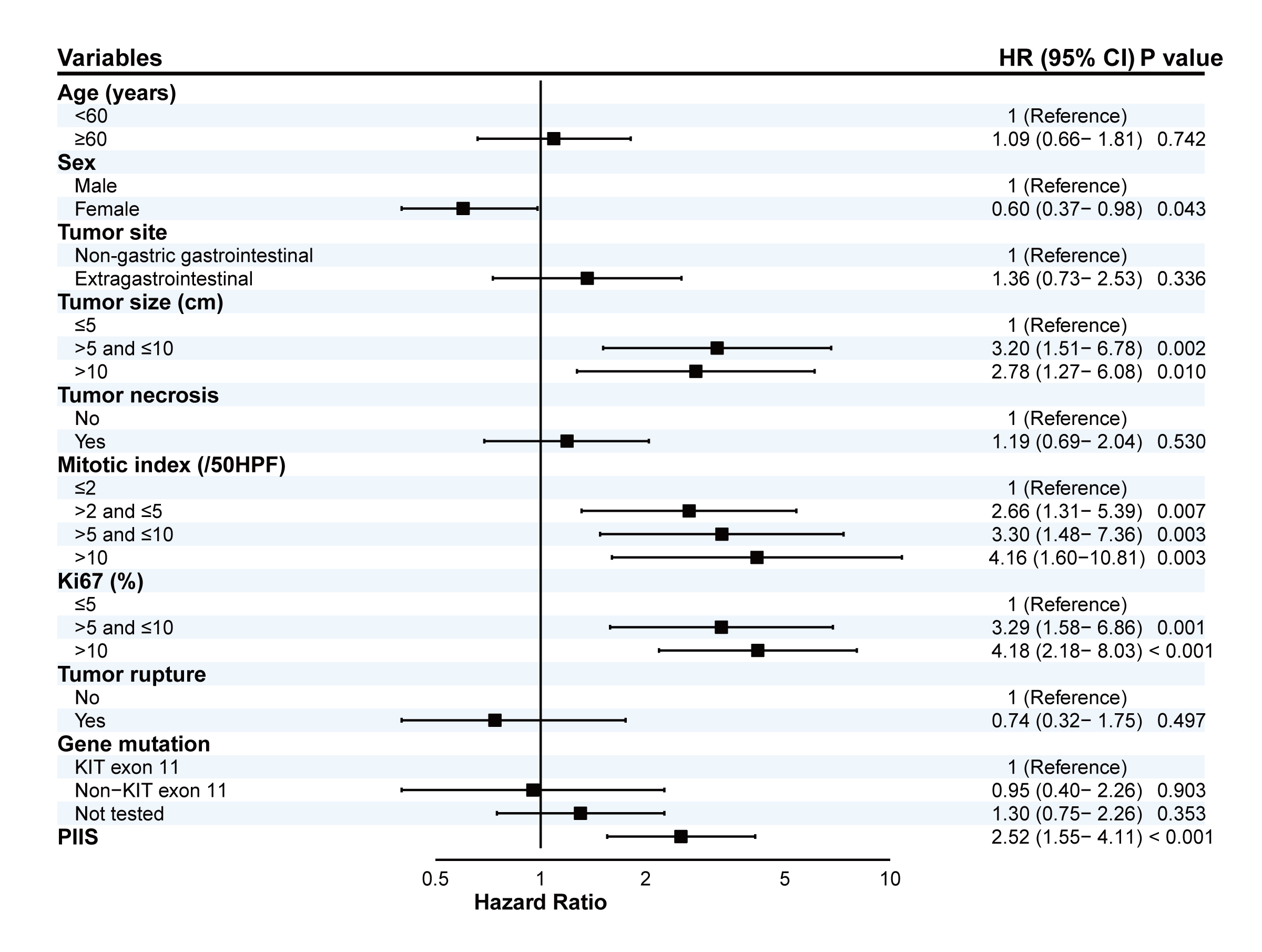
**

**Figure S12. Multivariable Cox regression analysis for recurrence-free survival (RFS).** The black squares represent the estimated HRs, and the horizontal lines represent the 95% CIs.

**
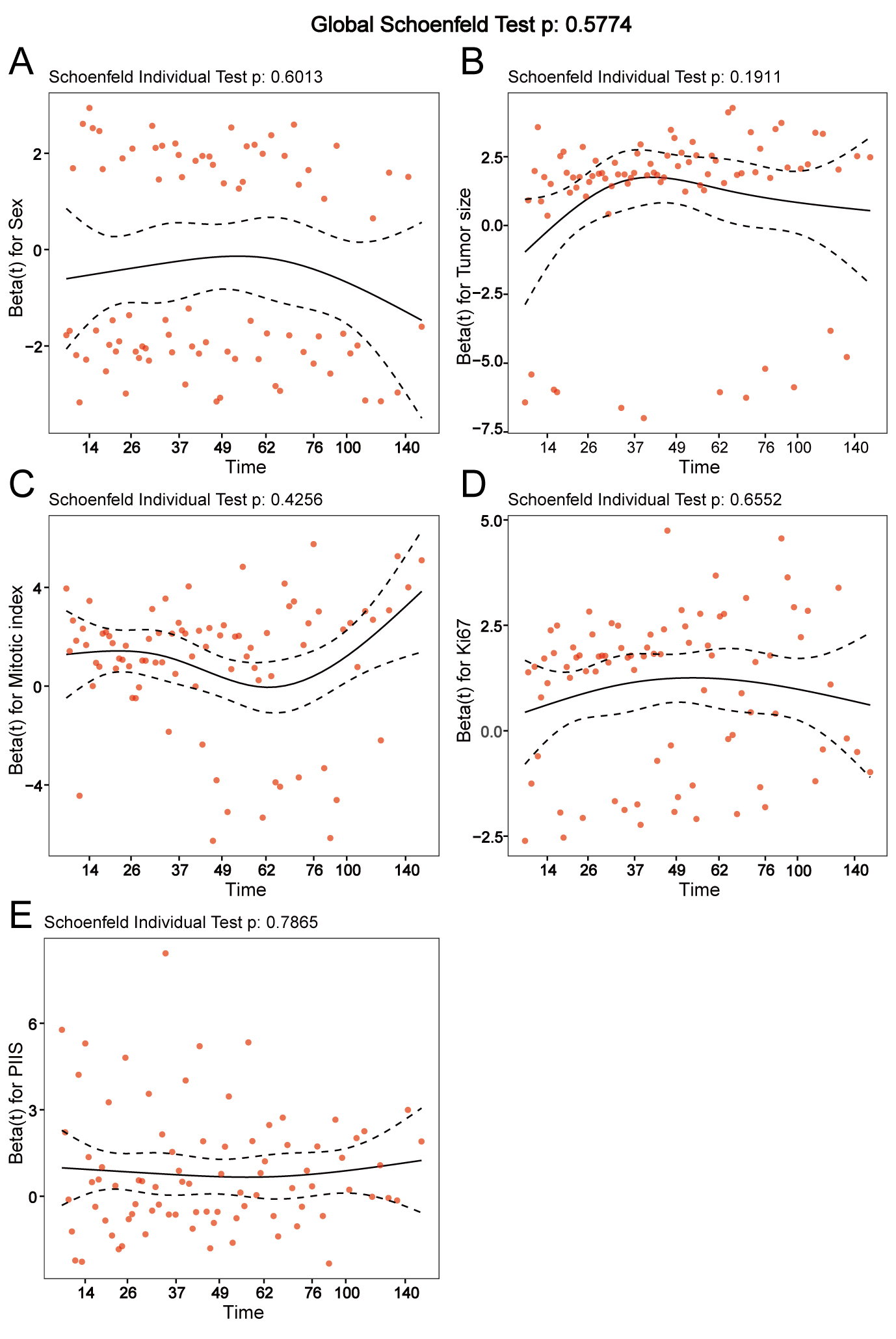
**

**Figure S13. Proportional hazards assumption validation.** Scaled Schoenfeld residuals plotted against time for (A) sex, (B) tumor size, (C) mitotic index, (D) Ki67 index, and (E) PIIS.

**
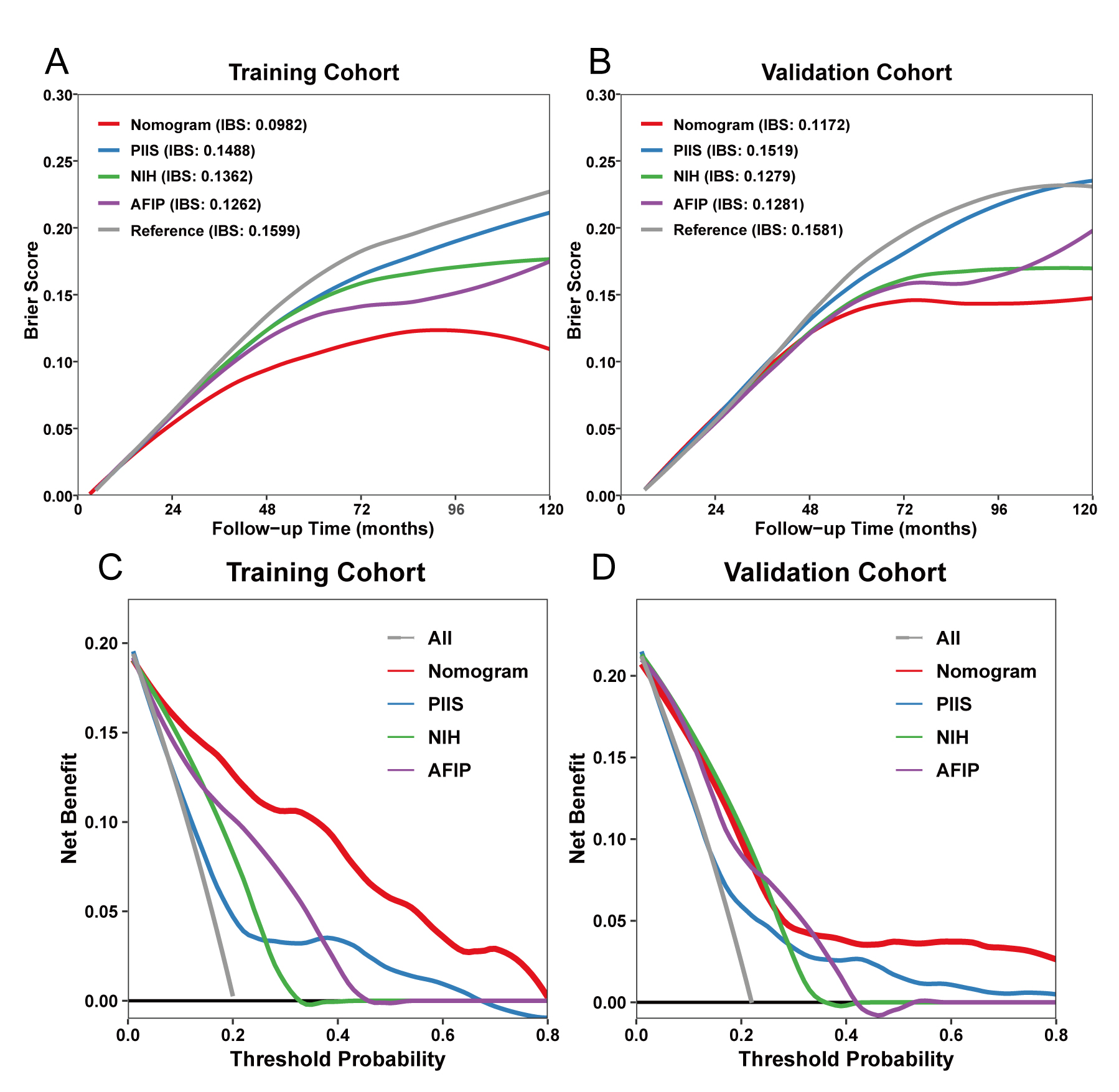
**

**Figure S14. Comparison of predictive performance and clinical utility among the nomogram, PIIS, and conventional risk stratification systems in the training and validation cohorts.** (A, B) Time-dependent Brier score curves. (C, D) Decision curve analysis (DCA).

**
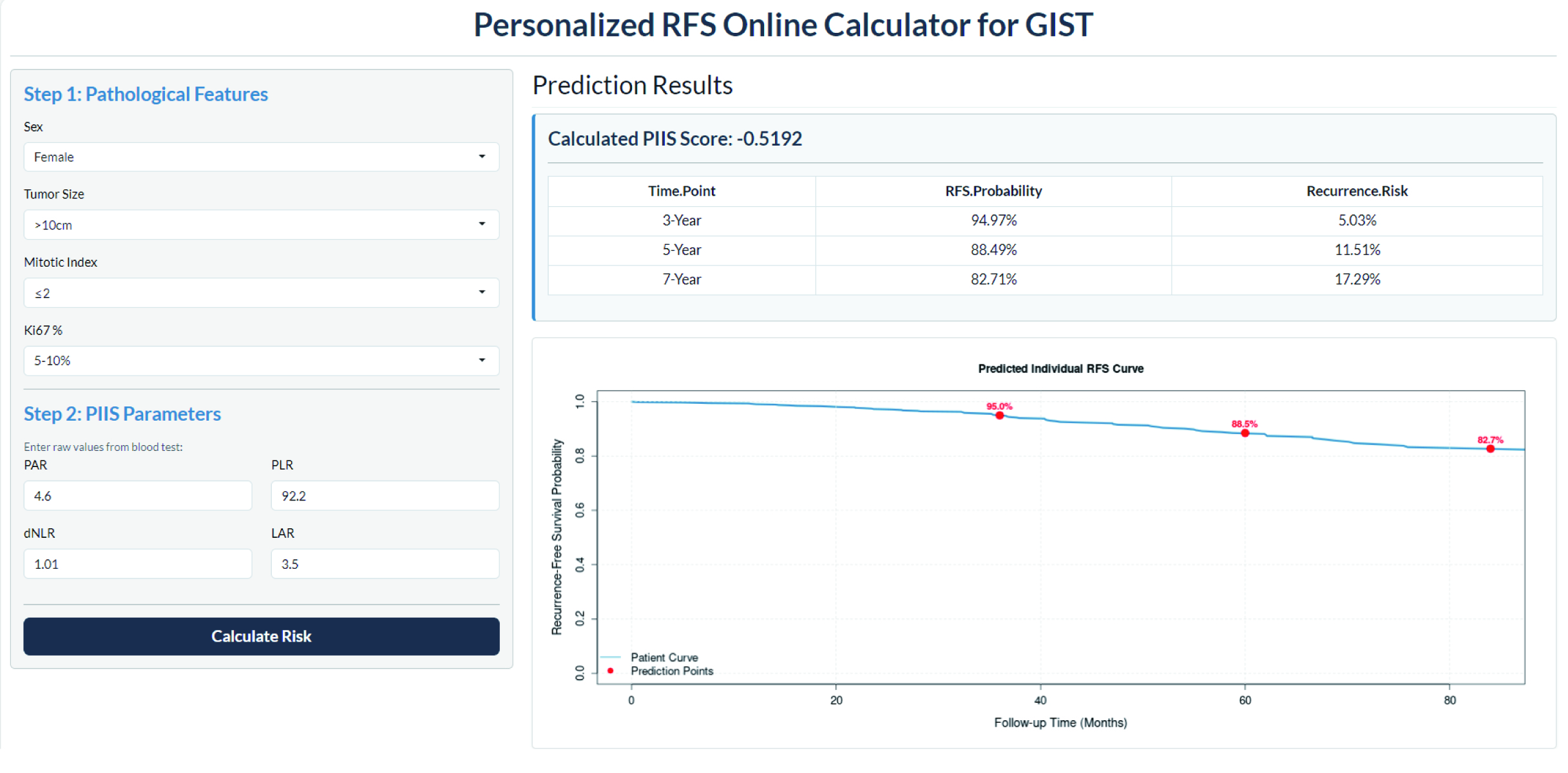
**

**Figure S15. Interface of the personalized online recurrence-free survival (RFS) calculator for GIST patients.** The online tool incorporates pathological features (sex, tumor size, mitotic index, Ki67) and inflammatory parameters (PAR, PLR, dNLR, LAR). Outputs include the PIIS score, 3-, 5-, and 7-year recurrence probabilities.

**
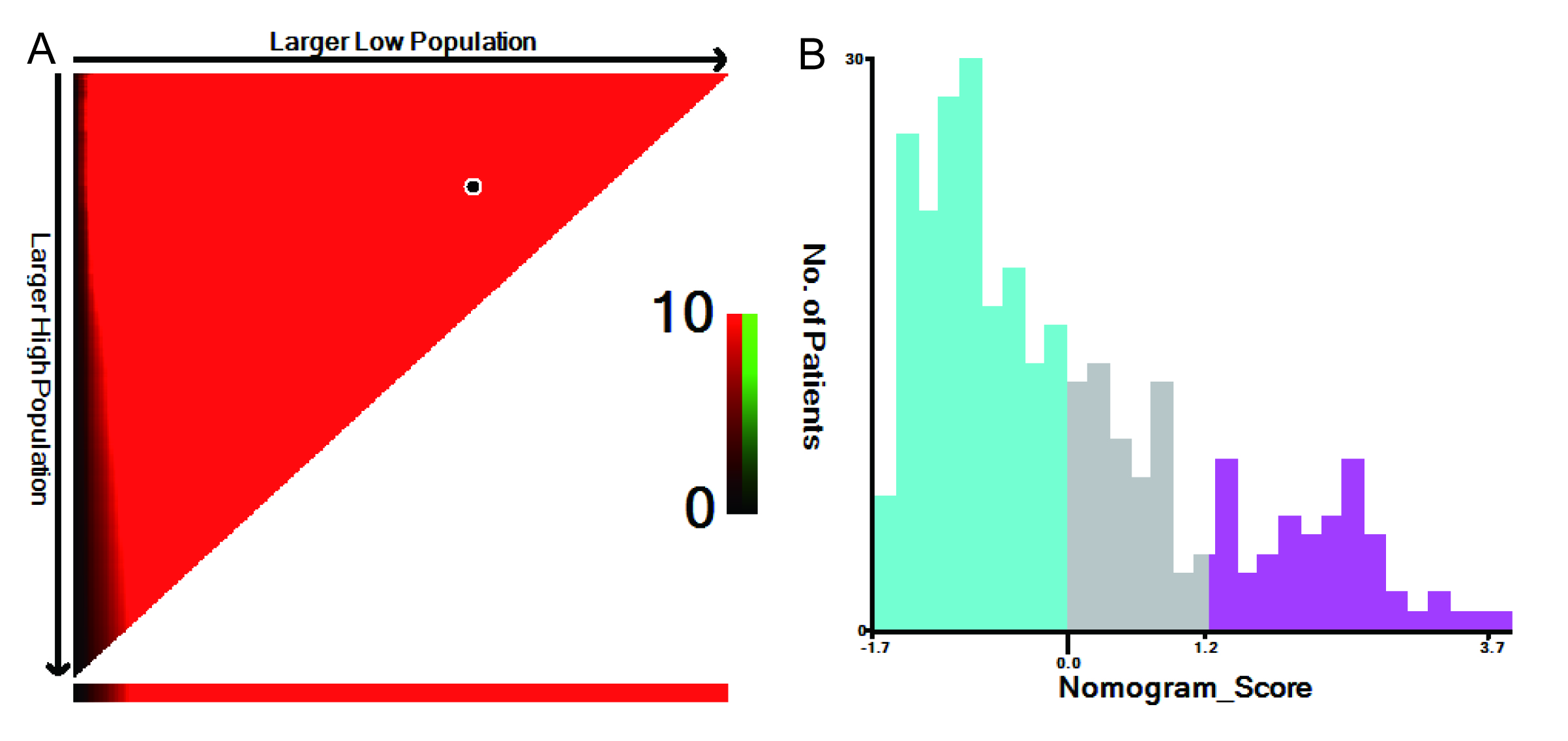
**

**Figure S16. Determination of optimal nomogram score cutoffs.** (A) X-tile plot showing association strength at various divisions. (B) Distribution of patients into low-, medium-, and high-risk groups based on optimal cutoffs (0.03 and 1.23) in the training cohort.

**
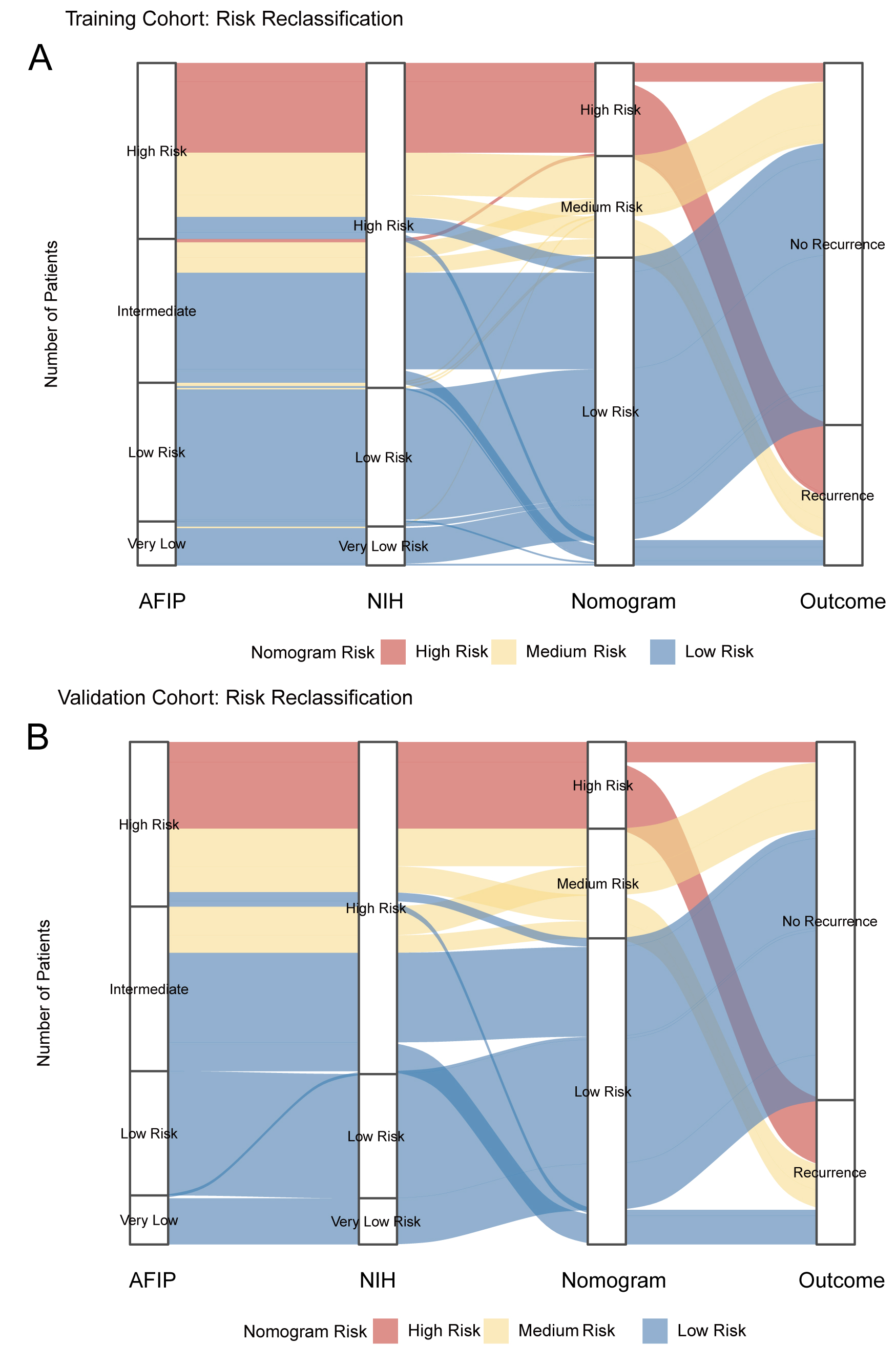
**

**Figure S17. Risk reclassification and outcomes.** Sankey diagrams illustrating patient migration from AFIP/mNIH risk categories to nomogram-defined categories in the (A) training and (B) validation cohorts.

**
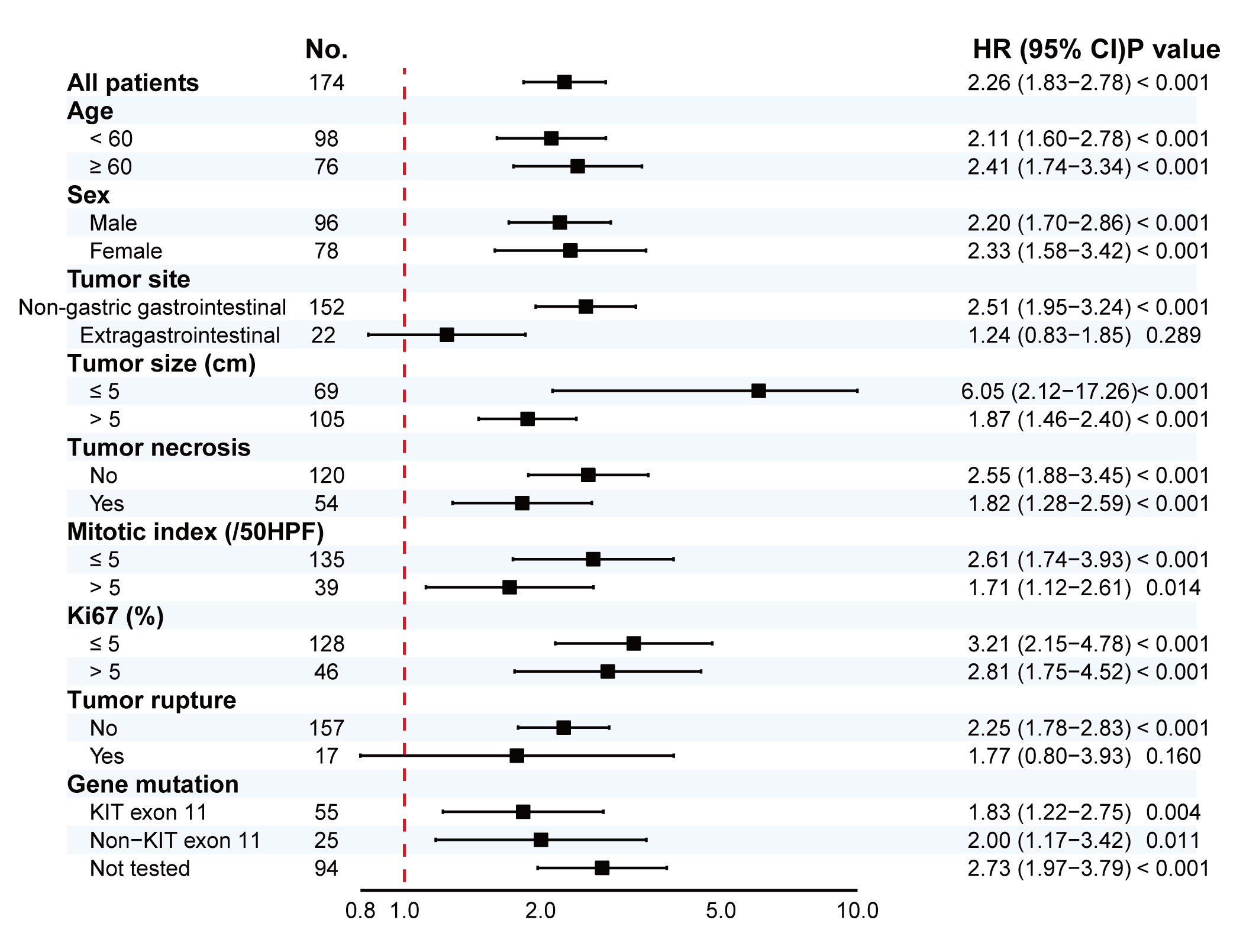
**

**Figure S18. Subgroup analysis of recurrence-free survival (RFS) according to the nomogram score in the validation cohort.** Black squares and horizontal lines represent the estimated HRs and their corresponding 95% CIs, respectively.

**
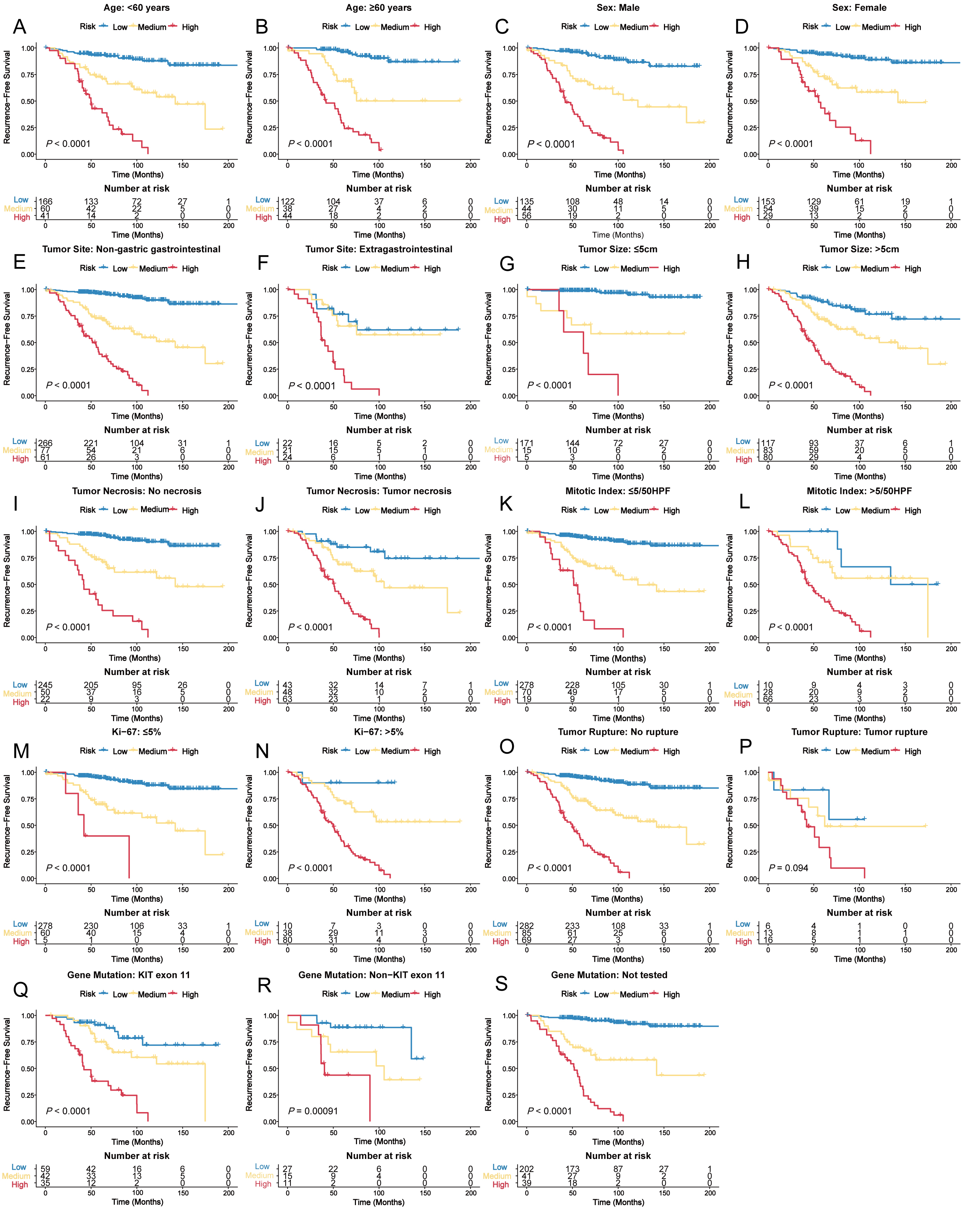
**

**Figure S19. Subgroup analysis of the nomogram-based risk stratification for recurrence-free survival (RFS).** (A–S) Kaplan–Meier curves for RFS according to the nomogram-defined risk categories (Low, Medium, and High risk), stratified by various clinicopathological characteristics.

**
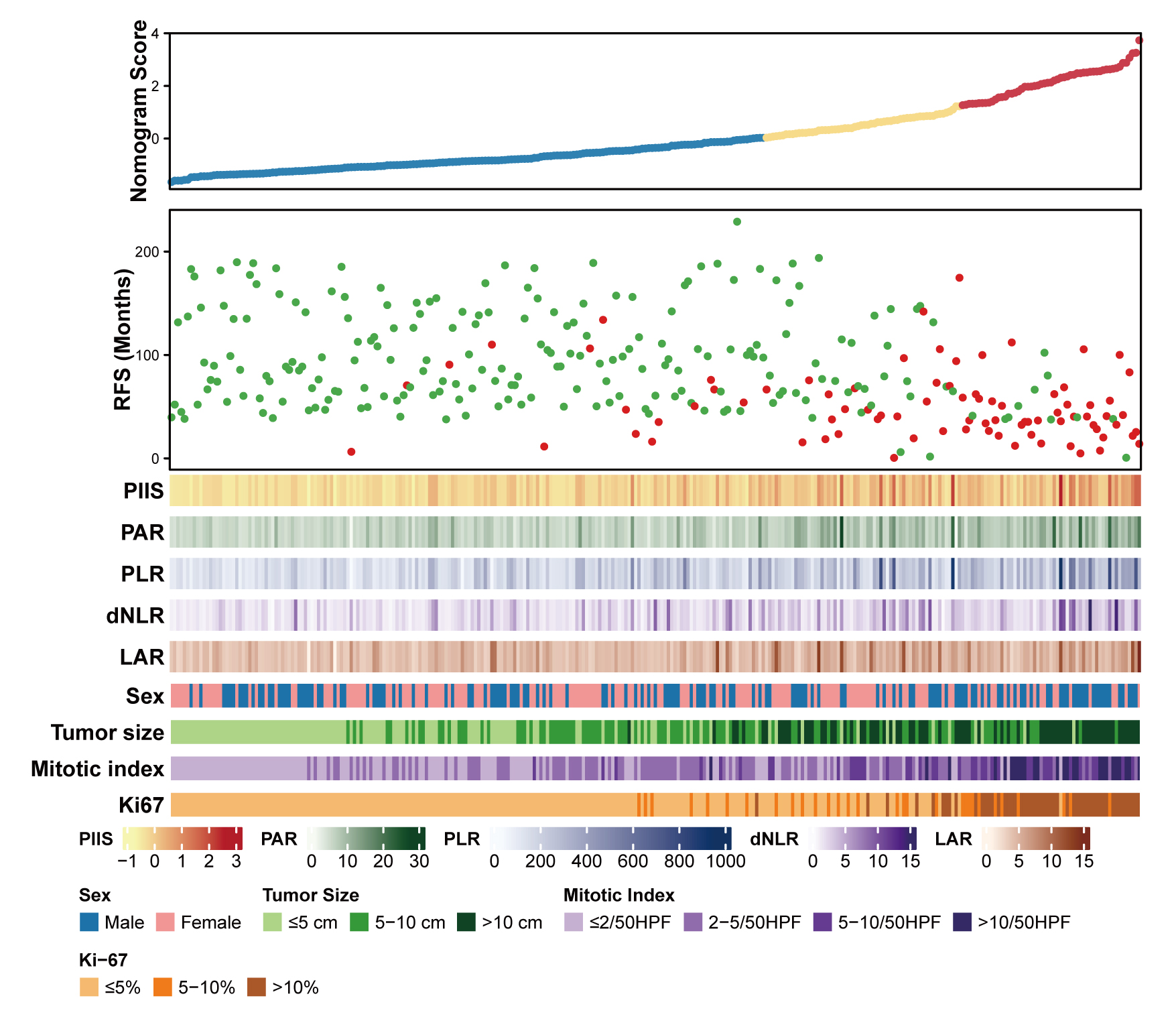
**

**Figure S20. Distribution of risk scores and clinicopathological features in the training cohort.** Top: Patients ranked by ascending nomogram scores, colored by risk category (Low, Medium, and High). Middle: Corresponding recurrence status and follow-up duration, with red and green dots representing events and censored cases, respectively. Bottom: Heatmap illustrating the distribution of individual prognostic features for each patient.

**
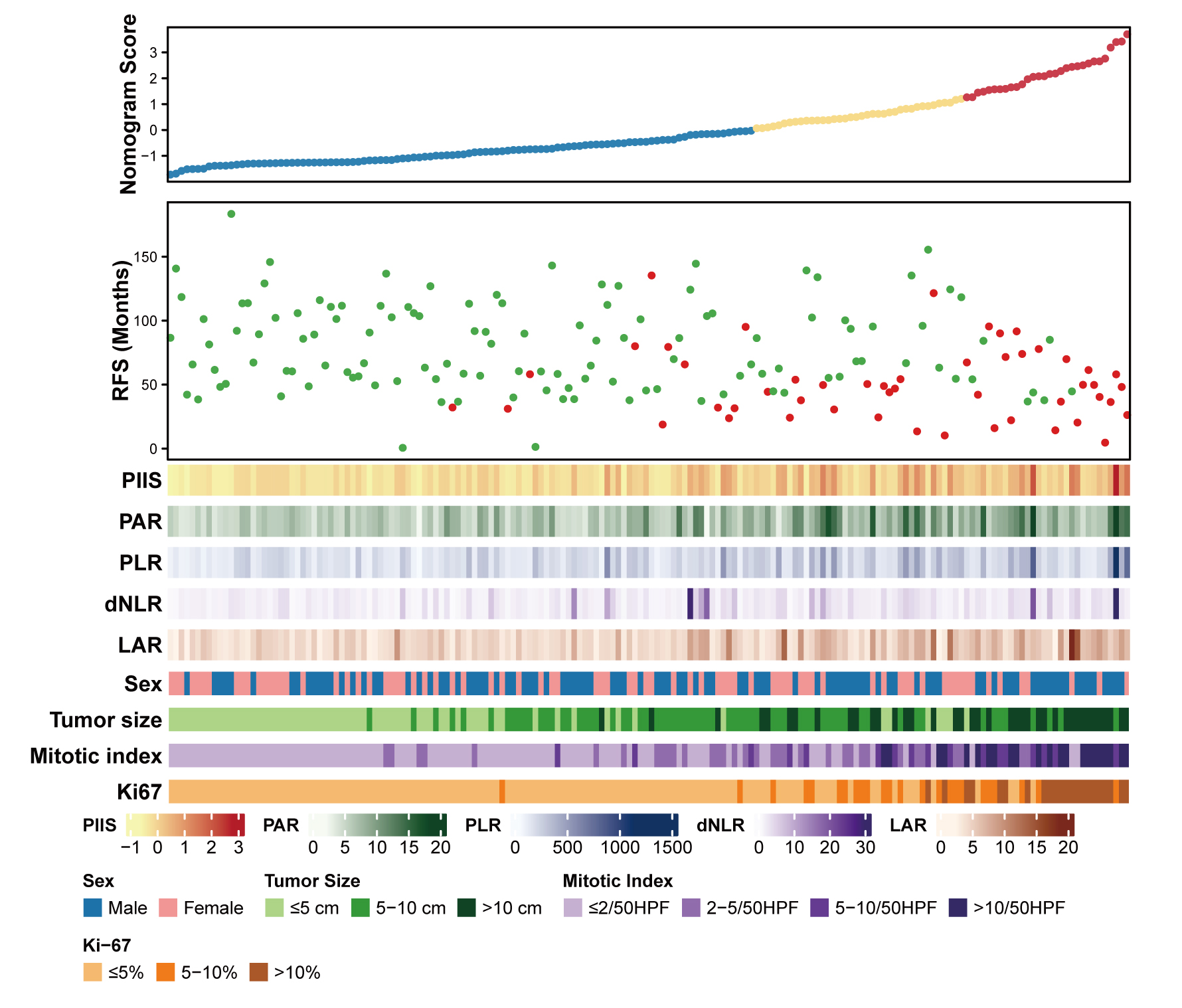
**

**Figure S21. Distribution of risk scores and clinicopathological features in the validation cohort.** Top: Patients ranked by ascending nomogram scores, colored by risk category (Low, Medium, and High). Middle: Corresponding recurrence status and follow-up duration, with red and green dots representing events and censored cases, respectively. Bottom: Heatmap illustrating the distribution of individual prognostic features for each patient.

**Supplementary Tables**

**Table S1** The C-index of each model in the training and validation cohorts

| **Models** | **Recurrence-free survival** | |
| --- | --- | --- |
|  | **C-index (95% CI)** | ***P*** |
| **Training cohort** | | |
| Integrated nomogram | 0.839 (0.798-0.879) | Ref |
| PIIS | 0.658 (0.595-0.721) | **<0.001** |
| Modified NIH criteria | 0.679 (0.642-0.717) | **<0.001** |
| AFIP criteria | 0.748 (0.698-0.798) | **<0.001** |
| **Validation cohort** | | |
| Integrated nomogram | 0.795 (0.740-0.851) | Ref |
| PIIS | 0.616 (0.525-0.707) | **<0.001** |
| Modified NIH criteria | 0.693 (0.654-0.732) | **<0.001** |
| AFIP criteria | 0.754 (0.700-0.809) | **0.033** |

The bold values indicate statistically significant P-values (P<0.05).

**Table S2** Akaike and Bayesian information criterion values for the integrated nomograms compared with traditional models

| **Models** | **Integrated nomograms** | **PIIS** | **Modified NIH criteria** | **AFIP criteria** |
| --- | --- | --- | --- | --- |
| **Training cohort** | | | | |
| AIC | 739.26 | 843.90 | 811.53 | 791.88 |
| BIC | 742.95 | 847.59 | 818.92 | 802.96 |
| **Validation cohort** | | | | |
| AIC | 406.47 | 454.67 | 419.78 | 409.14 |
| BIC | 409.63 | 457.83 | 426.10 | 418.62 |

Abbreviations: AIC, Akaike information criterion; BIC, Bayesian information criterion.
